# *Helicobacter pylori* Eradication Causes Perturbation of the Human Gut Microbiome in Young Adults

**DOI:** 10.1371/journal.pone.0151893

**Published:** 2016-03-18

**Authors:** Theresa Wan-Chen Yap, Han-Ming Gan, Yin-Peng Lee, Alex Hwong-Ruey Leow, Ahmad Najib Azmi, Fritz Francois, Guillermo I. Perez-Perez, Mun-Fai Loke, Khean-Lee Goh, Jamuna Vadivelu

**Affiliations:** 1 Department of Medical Microbiology, Faculty of Medicine, University of Malaya, 50603, Kuala Lumpur, Malaysia; 2 School of Science, Monash University Malaysia, 47500, Bandar Sunway, Selangor, Malaysia; 3 Monash University Malaysia Genomics Facility, Monash University Malaysia, 47500, Bandar Sunway, Selangor, Malaysia; 4 Department of Medicine, Faculty of Medicine, University of Malaya, 50603, Kuala Lumpur, Malaysia; 5 Faculty of Medicine and Health Sciences, Universiti Sains Islam Malaysia, 55100, Kuala Lumpur, Malaysia; 6 New York University Cancer Institute, New York, NY, 10016, United States of America; 7 Department of Medicine, New York University School of Medicine, New York, NY 10016, United States of America; 8 Department of Microbiology, New York University School of Medicine, New York, NY, 10016, United States of America; National Cancer Institute, UNITED STATES

## Abstract

**Background:**

Accumulating evidence shows that *Helicobacter pylori* protects against some metabolic and immunological diseases in which the development of these diseases coincide with temporal or permanent dysbiosis. The aim of this study was to assess the effect of *H*. *pylori* eradication on the human gut microbiome.

**Methods:**

As part of the currently on-going ESSAY (Eradication Study in Stable Adults/Youths) study, we collected stool samples from 17 *H*. *pylori*-positive young adult (18–30 years-old) volunteers. The same cohort was followed up 6, 12 and 18 months-post *H*. *pylori* eradication. The impact of *H*. *pylori* on the human gut microbiome pre- and post-eradication was investigated using high throughput 16S rRNA gene (V3-V4 region) sequencing using the Illumina Miseq followed by data analysis using Qiime pipeline.

**Results:**

We compared the composition and diversity of bacterial communities in the fecal microbiome of the *H*. *pylori*-positive volunteers, before and after *H*. *pyl*ori eradication therapy. The 16S rRNA gene was sequenced at an average of 150,000–170,000 reads/sample. The microbial diversity were similar pre- and post-*H*. *pylori* eradication with no significant differences in richness and evenness of bacterial species. Despite that the general profile of the gut microbiome was similar pre- and post-eradication, some changes in the bacterial communities at the phylum and genus levels were notable, particularly the decrease in relative abundance of *Bacterioidetes* and corresponding increase in *Firmicutes* after *H*. *pylori* eradication. The significant increase of short-chain fatty acids (SCFA)-producing bacteria genera could also be associated with increased risk of metabolic disorders.

**Conclusions:**

Our preliminary stool metagenomics study shows that eradication of *H*. *pylori* caused perturbation of the gut microbiome and may indirectly affect the health of human. Clinicians should be aware of the effect of broad spectrum antibiotics used in *H*. *pylori* eradication regimen and be cautious in the clinical management of *H*. *pylori* infection, particularly in immunocompromised patients.

## Introduction

The human body routinely harbors approximately 100 trillion bacteria which outnumber our human cells by 10 to 1 [1]. This bacterial population constitutes the microbiota and the majority of them living in the lower part of gastrointestinal tract. The human gut microbiota is an intricate and dynamic ecosystem that has coevolved with human for millions of years [2]. It has been regarded as a metabolically active “organ” located within the human gastrointestinal tract [3, 4] as it has developed metabolic traits that complement host’s metabolism [5–8]. A healthy gastrointestinal system relies on a balanced commensal biota to regulate processes such as energy metabolism [9–12], elimination of pathogens [13–15] and influences the signaling pathways that range from modulation of the mucosal immune response [16] to development of metabolic diseases [9–11, 17, 18]. Accumulating evidence suggests that dysbiosis, or an abnormal microbiota, has been associated with an increasingly long list of diseases, including inflammatory bowel disease, obesity, and atopic diseases such as eczema and asthma [13, 19].

*Helicobacter pylori* is a Gram-negative, spiral-shaped, microaerophilic bacterium that colonizes the gastric mucosa of humans and non-human primates naturally. More than half of the world’s population is infected with *H*. *pylori* and its prevalence is as high as 80% in some populations [20]. In a minority of those infected, *H*. *pylori* can cause peptic ulcers (10%), gastric cancer (1–2%), and rarely mucosa-associated lymphoid tissue (MALT) lymphoma [21]. *H*. *pylori* is believed to colonize the stomach of humans at least since the initial migration of our ancestors from East Africa approximately 60,000 years ago [22]. It has been postulated that *H*. *pylori* may be part of the human indigenous microbiome. However, due to socioeconomic development, modern hygienic practices and the advent of the antibiotics, the human gut microbiota is changing and *H*. *pylori* is gradually disappearing. There is an increasing number of epidemiological and experimental evidence for the protective effect of *H*. *pylori* infection on the development of obesity [23–25], childhood asthma [26], allergies [27], inflammatory bowel diseases [28], in which the development of these diseases coincide with either temporal or permanent dysbiosis.

Thus, in this study, we hypothesize that the eradication of *H*. *pylori* may cause perturbation of the GI microbiome, which can indirectly affect the health of human host. We investigated the effect of *H*. *pylori* eradication on the human gut microbiome using *16S rRNA* gene amplicon metagenomics sequencing up to 18 months post-eradication.

## Materials and Methods

### Study Population

Our study is part of the currently on-going ESSAY (Eradication Study in Stable Adults/Youths) study in Malaysia, New York and an European Center. In Malaysia, it was conducted at the University of Malaya Medical Centre (UMMC) between June 2012 and May 2014. Healthy young adults between the ages of 18–30 years old were first screened to assess study eligibility. The exclusion criteria for the study were diabetes, hyper or hypothyroidism, prior gastric or bariatric surgery, prior documented treatment of *H*. *pylori*, antibiotic use within 4 weeks of enrollment, steroid or other immunomodulating drugs use within 4 weeks of enrollment, recent vaccination and Charlson weighed comorbidity index <2. The study protocol was reviewed and approved by Medical Ethics Committee at UMMC (Ref No. 877.1). Written informed consent was obtained from qualified volunteers prior to study participation. *H*. *pylori*’s status of the qualified candidates was determined as previously described [29].

### Sample Collection

As we reported earlier, 57 (9.9%) of the 573 volunteers screened in the ESSAY study were tested positive for *H*. *pylori* using both non-radioactive ^13^C Urea Breath Test (UBT) and detection of anti-*H*. *pylori* antibodies and were considered as *H*. *pylori*-positive. However, only 32 agreed and consented to participate in the study [29]. Of these 32 *H*. *pylori* positive volunteers, we only managed to collect stool samples from 17 of them. Treatment with a 7-day twice daily regimen and a proton pump inhibitor as current standard of care (amoxicillin 1000 mg, clarithromycin 500 mg, and pantoprazole 40 mg) was given to the volunteers and approximately 6 weeks after completing the treatment protocol, *H*. *pylori* eradication were ascertained using UBT. The volunteers were subsequently followed up at 6, 12, and 18 months post-*H*. *pylori* eradication. Stool samples were collected during each visit and frozen immediately at -80°C until DNA extraction.

### Nucleic Acid Extraction from Stool Samples

Nucleic acid extraction was done using MoBio PowerSoil DNA Isolation Kit (Mo Bio Laboratories, Carlsbad, CA). The DNA extraction protocol was adopted from Section 7.9 Specimen Processing For Extraction of Bacterial Genomic DNA taken from Manual of Procedures for Human Microbiome Project: Core Microbiome Sampling Protocol A (HMP Protocol #07–001), Version 12.0 (http://www.hmpdacc.org/tools_protocols/tools_protocols.php) with slight modification [30]. Instead of pre-processing the stool specimen with MoBio lysis buffer, approximately 100–200 mg of stool sample was added directly into PowerBead Tubes and vortexed to dispense the sample. The subsequent steps in the manufacturer’s protocol were followed accordingly.

### 16S rRNA Gene Amplification and Sequencing

The V3-V4 region of the bacterial *16S* rRNA gene sequences were amplified using the primer pair 338F* (5′-NNNNCCTACGGGAGGCAGCAG-3′) and 1061R (5′-GACTACHVGGGTATCTAATCC-3′) containing the complete Illumina adapter [31, 32].

Briefly, each 50 μL of polymerase chain reaction (PCR) reaction contains 10 ng of fecal genomic DNA as template, 25 μL NEBNext High-Fidelity 2x PCR Master Mix (New England Biolabs, Ipswich, MA) and 1 μL of 10 μM of each primer. PCR reactions were carried out using the following protocol: (1) for the stool samples, an initial denaturation step performed at 98°C for 30 sec followed by 30 cycles of denaturation (98°C, 10 s), annealing (60°C, 10 s) and extension (72°C, 30 sec), and a final elongation of 1 min at 72°C. PCR products ~600 bases in size were gel-excised and purified using QIAquick Gel Extraction Kit (QIAGEN, Hilden, Germany). The libraries were quantified using KAPA library quantification kit (KAPA Biosystems, Capetown, South Africa), normalized, pooled and sequenced (2 x 250 bp paired-end read setting) on the MiSeq (Illumina, San Diego, CA) located at the Monash University Malaysia Genomics Facility.

### Bioinformatics Analysis

#### Sequence pre-processing and quality filtering

Demultiplexing and generation of raw fastq files for each individual library was performed on-board by the MiSeq Reporter Software. The forward and reverse 16S primer sequence located at the 5’ end of the forward and reverse reads, respectively, were trimmed using FASTX-Toolkit [33]. The trimmed paired-end reads were subsequently overlapped using PEAR: Illumina Paired-End reAd mergeR (default setting) [34].

#### Analysis of quality filtered reads using Qiime

The merged paired-end reads were analyzed using the Quantitative Insights into Microbial Ecology (Qiime) [35] pipeline. To perform detection and clustering of 16S rRNAs, an open-reference Operational Taxonomic Units (OTUs) picking approach was used. *pick_open_reference_otus*.*py* is the primary interface for open-reference OTU picking in QIIME, and includes taxonomy assignment, sequence alignment, and tree-building steps. In this open-reference OTU picking process, reads were firstly clustered against a Greengenes 13_8 reference sequence collection [36] (available at http://qiime.org/home_static/dataFiles.html) through closed-reference OTUs picking. Subsequently, 0.1% of the reads which failed to hit the reference sequence collection were randomly subsampled and clustered de novo using UCLUST [37], with an OTU cluster defined at a sequence similarity of 97%. Each cluster centroid was then chosen as a “new reference sequence” for another round of closed-reference OTU picking. OTU assignments for read that failed to hit the reference database were picked by an additional round of de novo clustering. The PyNAST alignment algorithm [38] was used to align the OTU representative sequences against the Greengenes core reference alignment [39] with a minimum identity of 75%, and then a phylogenetic tree was built using FastTree [40]. Finally we generated a OTU table (*biom summarize-table*) for downstream diversity analysis by excluding the sequences that had failed to align by PyNAST. We also used the generated OTU table to summarize microbiome communities by taxonomic levels (by default: phylum, class, order, family, genus) based on different time-points (*summarize_taxa_through_plots*.*py*).

#### Statistical methods

The raw data of the taxonomy summary results (.txt file) were exported to SPSS software version 20.0 (SPSS Inc., Chicago, IL) for statistical analysis. The mean abundance in percentage (%) and the 95% confidence interval (95% CI) for the phyla of stool microbiome at different time-points were calculated. Parametric paired-samples t-test was performed to compare the genera of the stool microbiome between Baseline vs. 6 months, Baseline vs. 12 months, and Baseline vs. 18 months post-*H*. *pylori* eradication; a two-tailed p-value of < 0.05 was considered significant. Pearson’s Correlation Coefficient was also performed to investigate the relationship between the phylum *Bacteroidetes*-to-*Firmicutes* ratio and the Body Mass Index (BMI) of the subjects across different time-points; a two-tailed p-value of < 0.05 was considered significant.

#### Diversity analysis

We evaluated samples for alpha diversity (microbial diversity within samples) and beta diversity (community diversity between samples) analysis using Qiime. Alpha diversity analysis (*alpha_rarefaction*.*py*) involves rarefaction analysis by subsampling OTU table on the basis of a minimum rarefaction depth value that is chosen depending on the minimum number of sequences/sample obtained. For our study, the rarefaction depth value for the comparison of Baseline and 6 months post-eradication was set as 82,536, the rarefaction depth value for the comparison of Baseline and 12 months post-eradication was set as 84,177, whereas the rarefaction depth value for the comparison of Baseline and 18 months post-eradication was set as 84,333. The alpha diversity was then calculated using both “non-phylogeny-based” (observed species, chao1, Shannon index) and “phlogeny-based” (PD whole tree) matrices for each rarefied OTU table. We compared the alpha diversity between different groups (time-points) of the samples by non-parametric two-sample t-test (*compare_alpha_diversity*.*py*).

Beta diversity between our samples was calculated using the default beta diversity metrics of weighted and unweighted UniFrac [41] (*beta_diversity_through_plots*.*py*) on even subsampled OTU table. The resulting UniFrac distance matrices were used to perform Principal Coordinate Analysis (PCoA) to determine the similarity between groups of samples/time-points. The PCoA plots in three dimensions were visualized using the Emperor tool [42]. Non-parametric statistical analysis ANOSIM was performed via QIIME (*compare_categories*.*py—method anosim*) to test the statistical significance between different time-points (Baseline vs. 6 months post-eradication and Baseline vs. 12 months eradication).

## Results

### Demographics of the Study Cohort

Stool samples from 17 *H*. *pylori*-positive healthy young Malaysian with a mean age of 25 years were collected. The volunteers were followed up after they were given *H*. *pylori* eradication therapy. Only 17, 10, and 6 stool samples were successfully collected at 6, 12, and 18 months post-eradication, respectively. All the 16S rRNA sequences were deposited in MetaGenome Rapid Annotation using Subsystem Technology (MG-RAST) under the accession numbers as shown in Table 1.

**Table 1:** Accession number of the 16S rRNA sequences deposited in MG-RAST.

**Accession number**	**Time-point**	**Sample code**
4562320.3	Baseline	C002
4562322.3	Baseline	C003
4562324.3	Baseline	C005
4562326.3	Baseline	C008
4562328.3	Baseline	C009
4562330.3	Baseline	C017
4562332.3	Baseline	C019
4562334.3	Baseline	C020
4562340.3	Baseline	C033
4562342.3	Baseline	C034
4562344.3	Baseline	C037
4562346.3	Baseline	C039
4562348.3	Baseline	C041
4562350.3	Baseline	C042
4562352.3	Baseline	C048
**Accession number**	**Time-point**	**Sample code**
4562354.3	Baseline	C050
4562356.3	Baseline	C053
4562321.3	6 months post-eradication	C002
4562323.3	6 months post-eradication	C003
4562325.3	6 months post-eradication	C005
4562327.3	6 months post-eradication	C008
4562329.3	6 months post-eradication	C009
4562331.3	6 months post-eradication	C017
4562333.3	6 months post-eradication	C019
4562335.3	6 months post-eradication	C020
4562341.3	6 months post-eradication	C033
4562343.3	6 months post-eradication	C034
4562345.3	6 months post-eradication	C037
4562347.3	6 months post-eradication	C039
4562349.3	6 months post-eradication	C041
4562351.3	6 months post-eradication	C042
4562353.3	6 months post-eradication	C048
4562355.3	6 months post-eradication	C050
4562357.3	6 months post-eradication	C053
4626552.3	12 months post-eradication	C003
4626553.3	12 months post-eradication	C009
4626556.3	12 months post-eradication	C017
4626557.3	12 months post-eradication	C033
**Accession number**	**Time-point**	**Sample code**
4626558.3	12 months post-eradication	C034
4626560.3	12 months post-eradication	C037
4626562.3	12 months post-eradication	C039
4626564.3	12 months post-eradication	C041
4626567.3	12 months post-eradication	C048
4626568.3	12 months post-eradication	C053
4626559.3	18 months post-eradication	C034
4626561.3	18 months post-eradication	C037
4626563.3	18 months post-eradication	C039
4626565.3	18 months post-eradication	C041
4626566.3	18 months post-eradication	C042
4626569.3	18 months post-eradication	C053

### Characterization of Stool Microbiome

A total of 5,834,726 quality-filtered reads were obtained from Baseline vs. 6 months post-eradication group with an average of 171,610 ± 58,372 (standard deviation, SD) reads per sample, whilst, a total of 3,077,037 quality-filtered reads were obtained from Baseline vs. 12 months post-eradication group with an average of 153,852 ± 32,306 reads per sample. For Baseline vs. 18 months post-eradication group, a total of 2,000,505 quality-filtered reads with an average of 166,709 ± 69, 793 reads per sample were obtained. These reads were clustered into 45,875 unique OTUs at 97% sequence similarity in Baseline vs. 6 months post-eradication group with an average of 1349 OTUs per sample. In Baseline vs. 12 months post-eradication group, these reads were clustered into 31,351 unique OTUs at 97% sequence similarity with an average of 1568 OTUs per sample. In Baseline vs. 18 months post-eradication group, they were clustered into 28,157 unique OTUs at 97% sequence similarity with an average of 2346 OTUs per sample.

**Fig 1:**
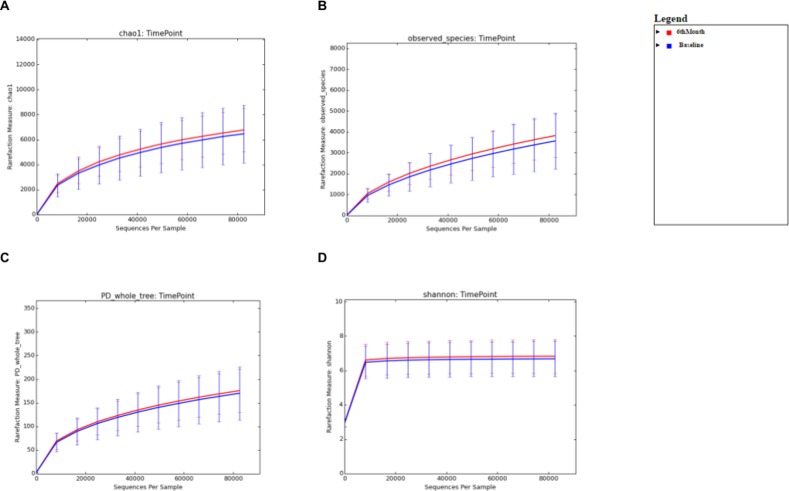
Alpha diversity analysis comparing between baseline and 6-months post-*H*. ***pylori* eradication.** Rarefraction curve for **A.** chao1, **B.** observed species, **C.** PD whole tree, and **D.** the Shannon index generated from alpha diversity analysis.

**Fig 2:**
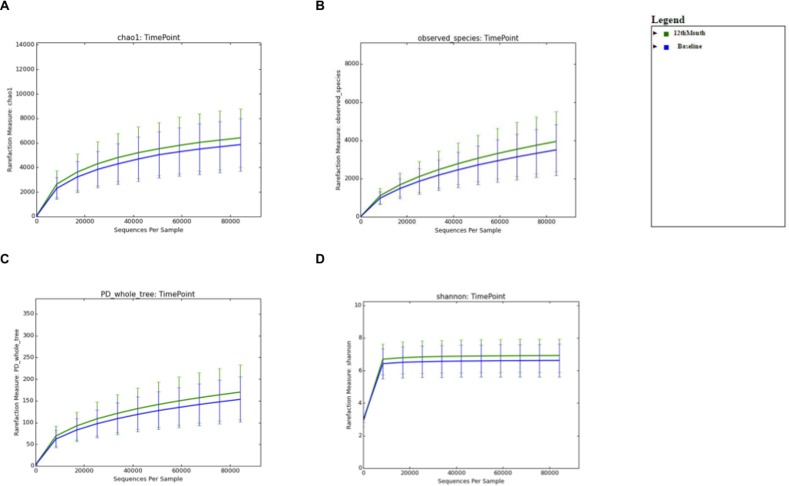
Alpha diversity analysis comparing between baseline and 12-months post-*H*. *pylori* eradication. Rarefraction curve for **A.** chao1, **B.** observed species, **C.** PD whole tree, and **D.** the Shannon index generated from alpha diversity analysis.

**Fig 3:**
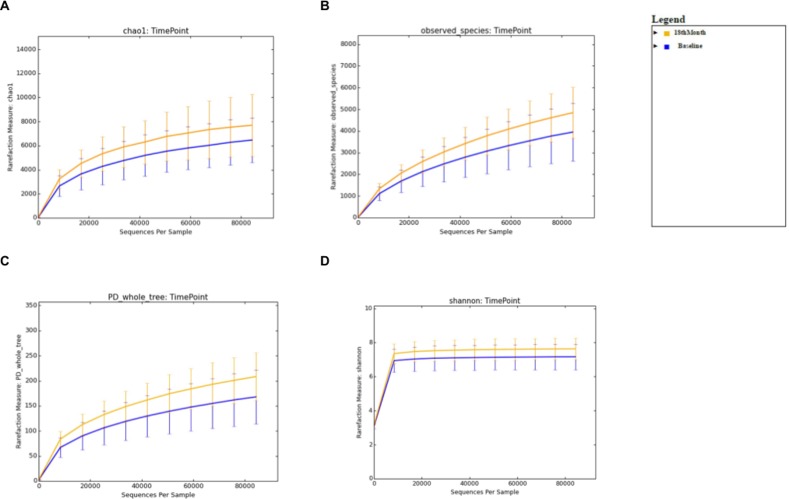
Alpha diversity analysis comparing between baseline and 18-months post-*H*. *pylori* eradication. Rarefraction curve for **A.** chao1, **B.** observed species, **C.** PD whole tree, and **D.** the Shannon index generated from alpha diversity analysis.

For the comparison of microbial biodiversity within Baseline and 6 months post-eradication stool samples, alpha diversity analysis was performed after rarefaction to 82,536 sequences/sample (minimum sampling depth). For the comparison of microbial diversity within Baseline and 12 months post-eradication stool samples, alpha diversity analysis was performed after rarefaction to 84,177 sequences/sample. Whereas, for comparison of microbial diversity within Baseline and 18 months post-eradication stool samples, alpha diversity analysis was performed after rarefaction to 84,333 sequences/sample. We used several “phylogeny-based” and “non-phylogeny-based” matrices to calculate alpha diversity, including PD whole tree, chao1, observed species for microbial richness and the Shannon index for microbial evenness. When Baseline samples was compared with 6 months post-eradication samples, the rarefaction curves generated for all four matrices showed that the stool microbiome in 6 months-post eradication samples demonstrated greater diversity than Baseline samples (Fig 1). However, non-parametric two-sample t-test performed on the four matrices showed that there was no significant difference of the microbial diversity within baseline and 6 months post-eradication stool samples (p>0.05). Similarly, when Baseline samples was compared with 12 or 18 months post-eradication samples, the rarefaction curves generated for all four matrices showed that the stool microbiome in 12 and 18 months-post eradication samples demonstrated greater diversity than Baseline samples (Figs 2 and 3). However, non-parametric two-sample t-test performed on the four matrices showed that the microbial biodiversity within baseline and 12 months post-eradication as well as within baseline and 18 months post-eradication stool samples also did not differ significantly (p>0.05).

The PCoA plots generated from both weighted and unweighted UniFrac distance metrics in beta diversity analysis for Baseline vs. 6 months post-eradication, Baseline vs. 12 months post-eradication and Baseline vs. 18 months post-eradication did not show distinct clustering between the time-points (S1 & S2 Figs). Non-parametric statistical test analysis of similarity (ANOSIM) also showed that the differences in bacterial communities between the time-points were not significant (p>0.05).

**Fig 4:**
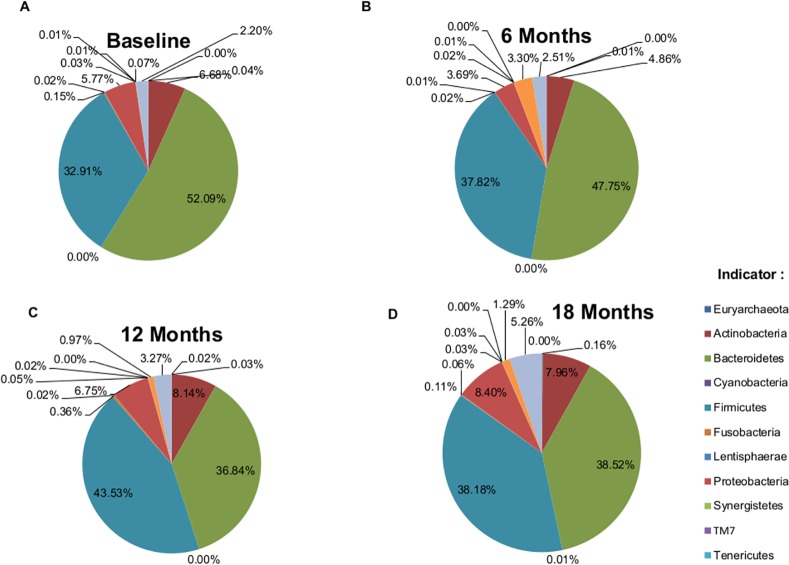
Relative abundance of phyla. Relative abundance of phyla at **A.** Baseline, **B.** 6 months-post eradication, **C.** 12 months-post eradication, and **D.** 18 months-post eradication.

**Fig 5:**
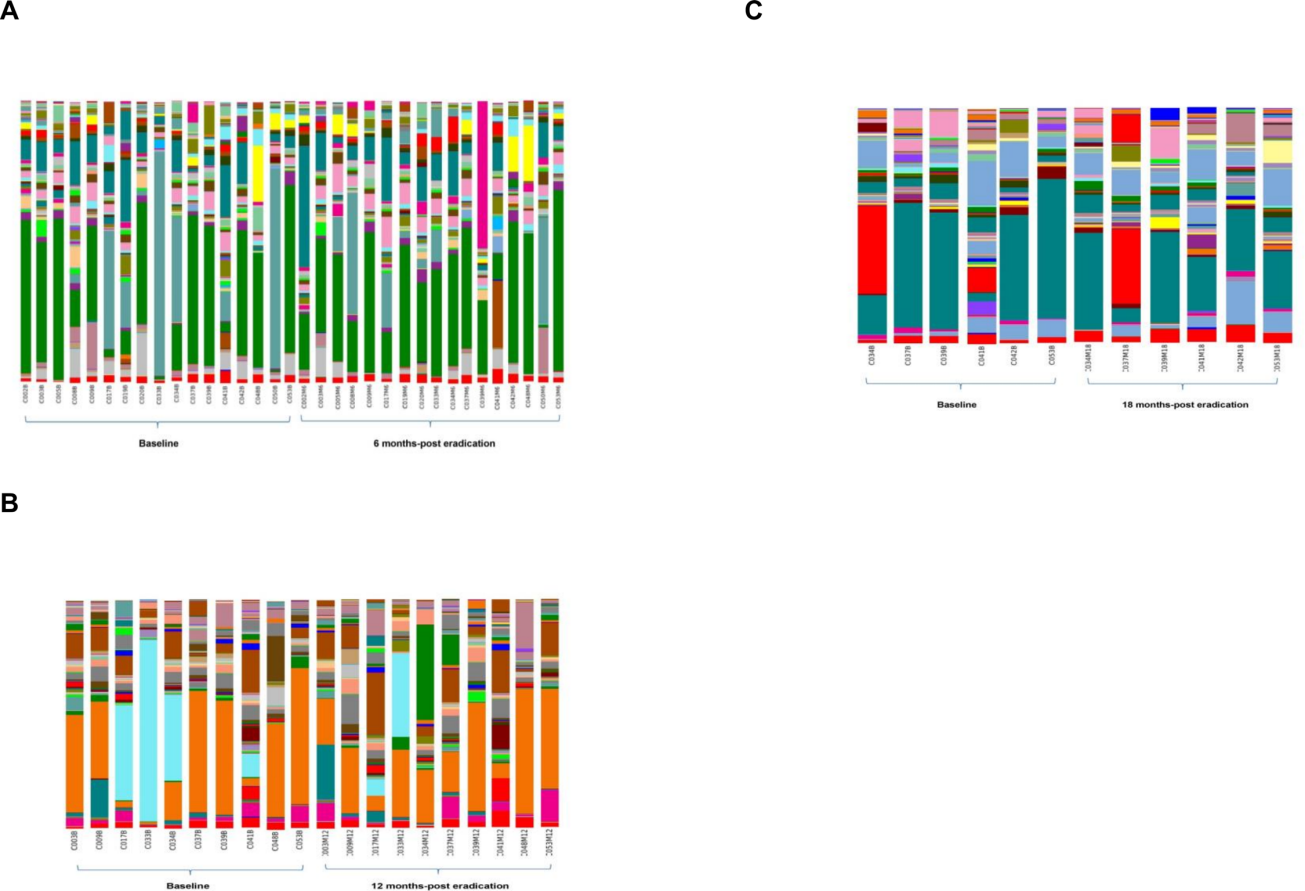
Relative abundance of genera. Comparison of the relative abundance of genera between **A.** Baseline vs. 6 months-post eradication, **B.** Baseline vs. 12 months-post eradication, and **C. B**aseline vs. 18 months-post eradication.

The taxonomy summary of the phyla and genera of stool microbiome in healthy young Malaysian adults pre- and post-eradication of *H*. *pylori* were summarized in Fig 4 and Fig 5. Before the eradication *H*. *pylori*, the most abundant phyla were *Bacteroidetes* (52.09%; 95% CI, 44.85%-60.07%), *Firmicutes* (32.91%; 95% CI, 26.67%-39.06%), *Actinobacteria* (6.68%; 95% CI, 4.03%-9.68%), and *Proteobacteria* (5.77%; 95% CI, 3.94%-8.03%) (Fig 1A). At 6 months post-*H*. *pylori* eradication, the relative abundance of *Bacteroidetes* decreased to 47.82% (95% CI, 42.24%-52.94%) and *Firmicutes* increased to 37.82% (95% CI, 32.19%-43.71%), as compared to Baseline. In addition, the relative abundance of both phyla *Actinobacteria* (4.86%; 95% CI, 2.65%-7.27%), and *Proteobacteria* (3.69%; 95% CI, 2.58%-4.92%) also reduced 6 months post-eradication. Interestingly, we also observed that the relative abundance of *Verrucomicrobia* increased markedly, from 0.07% (95% CI, 0.01%-0.17%) at Baseline to 3.30% (95% CI, 0.08%-9.53%) 6 months post-eradication (Fig 1B). At 12 months post-*H*. *pylori* eradication, *Firmicutes* (43.53%; 95% CI, 31.66%-54.29%) replaced *Bacteroidetes* (36.84%; 95% CI, 26.45%-49.26%), as the most abundant phylum in the stool microbiome. Similar patterns were observed in *Actinobacteria* (8.14%; 95% CI, 3.34%-14.48%), *Proteobacteria* (6.75%; 95% CI, 2.93%-12.39%), and *Fusobacteria* (0.36%; 95% CI, 0%-1.07%) in which their relative abundances higher than Baseline. The relative abundance of *Verrucromicrobia* (0.97%; 95% CI, 0.2%-2.1%), on the other hand, seems to be restoring to the Baseline level though its abundance was still higher than that of Baseline (Fig 1C). At 18 months-post *H*. *pylori* eradication, both *Bacteroidetes* and *Firmicutes* had the highest relative abundance which was approximately 38% for both phyla. Enrichment of the relative abundance of *Proteobacteria* (8.40%; 95% CI, 3.77%-13.03%) and *Actinobacteria* (7.96%; 95% CI, 3.11%-14.57%) were observed where their relative abundance increased to higher than Baseline, 6 months, and also 12 months post-eradication. The relative abundance of *Verrucomicrobia* (1.29%; 95% CI, 0%-3%) at 18 months-post eradication was still higher than Baseline whereas the relative abundance of *Fusobacteria* (0.11%; 95% CI, 0%-0.32%) had been restored to Baseline level (Fig 1D) (Table 2). The *Bacteroidetes*-to-*Firmicutes* ratio at Baseline, 6 months, 12 months, and 18 months were 8:5, 13:10, 5:6, and 1:1, respectively. Despite the observed trend in *Bacteroidetes*-to-*Firmicutes* ratio, these changes across time were not statistically significant (Paired-samples t-test, p≥0.05). The *Bacteroidetes*-to-*Firmicutes* ratio were also not significantly correlated with BMI of the volunteers across different time-points (Person’s correlation, p≥0.05).

**Table 2:** Comparison of Relative Abundance of Phyla of the Gut Microbiome Pre- and Post-*H*. *pylori* Eradication.

	**Mean relative abundance (%)**				**Comparison of the relative**	**Comparison of the relative**	**Comparison of the relative**
**Phylum**	(95% CI^*^)				**abundance at baseline and**	**abundance at baseline and**	**abundance at baseline and 18**
	**Baseline**	**6 Months-post**	**12 Months-post**	**18 Months-post**	**6 months post-eradication**	**12 months post-eradication**	**months post-eradication**
		**eradication**	**eradication**	**eradication**	**(p-value)^a^**	**(p-value)^a^**	**(p-value)^a^**
Firmicutes	32.91	37.82	43.53	38.17	0.31	0.12	0.42
	(26.67–39.06)	(32.19–43.71)	(31.66–54.29)	(31.53–44.66)			
Bacteroidetes	52.09	47.75	36.84	38.52	0.41	**0.02**	0.13
	(44.85–60.07)	(42.24–52.94)	(26.45–49.26)	(33.22–43.77)			
Actinobacteria	6.68	4.86	8.14	7.96	0.41	0.54	0.36
	(4.03–9.68)	(2.65–7.27)	(3.34–14.48)	(3.11–14.57)			
Proteobacteria	5.77	3.69	6.75	8.40	0.17	0.85	0.69
	(3.94–8.03)	(2.58–4.92)	(2.93–12.39)	(3.77–13.03)			
Fusobacteria	0.15	0.02	0.36	0.11	0.23	0.77	0.35
	(0–0.41)	(0–0.05)	(0–1.07)	(0–0.32)			
Verrucomicrobia	0.07	3.30	0.97	1.29	0.31	0.12	0.21
	(0.01–0.17)	(0.08–9.53)	(0.20–2.11)	(0–3)			
Euryarcheota	0.04	0.01	0.03	0.16	0.21	0.73	0.37
	(0–0.11)	(0–0.02)	(0–0.07)	(0–0.48)			
Synergistetes	0.03	0.02	0.05	0.03	0.60	0.87	0.29
	(0–0.07)	(0–0.06)	(0–0.10)	(0–0.08)			
Lentisphaerae	0.02	0.01	0.02	0.06	0.30	0.41	0.36
	(0–0.04)	(0–0.02)	(0–0.05)	(0–0.16)			
	**Mean relative abundance (%)**				**Comparison of the relative**	**Comparison of the relative**	**Comparison of the relative**
**Phylum**	(95% CI^*^)				**abundance at baseline and**	**abundance at baseline and**	**abundance at baseline and 18**
	**Baseline**	**6 Months-post**	**12 Months-post**	**18 Months-post**	**6 months post-eradication**	**12 months post-eradication**	**months post-eradication**
		**eradication**	**eradication**	**eradication**	**(p-value)**^**a**^	**(p-value)**^**a**^	**(p-value)**^**a**^
TM7	0.01	0.01	0.02	0.03	0.05	0.3	**0.08**
	(0–0.01)	(0–0.01)	(0–0.04)	(0.01–0.05)			
Tenericutes	0.01	0.00	0.00	0.00	0.26	0.26	0.36
	(0–0.02)						
Cyanobacteria	0.00	0.00	0.00	0.01	0.39	0.24	0.10
				(0–0.02)			
Other	0.00	0.00	0.02	0.00	0.82	0.36	0.18
(bacteria)			(0–0.04)				
Other	2.2	2.51	3.27	5.26	0.23	0.17	0.05
(not assigned)	(1.70–2.70)	(2.05–3.06)	(2.23–4.57)	(4.02–6.42)			

p-values< 0.05 were indicated in bold.

*Bootstrapped 95% confidence interval (CI) was based on 1000 replicates.

^a^Paired-Samples T Test was used in comparing baseline and post-*H*. *pylori* eradication.

When the genera of stool microbiome between Baseline and 6 months post-*H*. *pylori* eradication group were compared, the relative abundance of *Anaerofustis*, *Phascolarctobacterium*, and *Ruminococcus* (Family: *Lachnospiraceae*) in the stool microbiome were found to have increased significantly whereas the relative abundance of an unnamed genus under Candidate Division TM7 phylum (Order & Family: unnamed)decreased significantly (Paired-samples t-test, p<0.05) (Table 3). The relative abundance of three genera in the stool microbiome changed significantly (p<0.05) when baseline was compared with 12 months post-eradication groups. The relative abundance of *Dialister* (p = 0.033) and *Helicobacter* (p = 0.041) increased while the relative abundance of *Agrobacterium* (p = 0.031) decreased 12 months post-*H*. *pylori* eradication (Table 4). *Agrobacterium* was detected in Baseline and 6 months post-eradication samples but disappeared in 12 and 18 months post-eradication samples. When the genera of stool microbiome between Baseline and 18 months post-*H*. *pylori* eradication group were compared, genus *Helicobacter* (p = 0.033) and another unnamed genus under Candidate Division TM7 phylum (Family: *Rs-045*) was found to have increased significantly (Table 5).

**Table 3:** Comparison of Relative Abundance of Genera of the Gut Microbiome at Baseline and 6 Months-Post *H*. *pylori* Eradication.

	Mean relative abundance		Comparison of relative abundance at
Genus	(95% CI*)		baseline and 6 months post-eradication
	Baseline	6 Months-Post Eradication	(p-value)^a^
*Anaerofustis*	7.70E-4%	1.87E-3%	0.016
	(1.81E-4%-1.43E-3%)	(9.17E-4%-2.91E-3%)	
*Phascolarctobacterium*	0.72%	1.76%	0.038
	(0.29%-1.26%)	(0.82%-3.05%)	
*Ruminococcus*	0.60%	1.25%	0.030
(Family: *Lachnospiraceae*)	(0.37%-0.89%)	(0.76%-1.88%)	
Unknown	0.01%	4.22E-3%	0.027
(Phylum: *TM7*;	(4.84E-3%-0.01%)	(2.54E-3%-5.82E-3%)	
Class: *TM7-3*)			

*Bootstrapped 95% confidence interval (CI) was based on 1000 replicates.

^a^Paired-Samples T Test was used in comparing baseline and post-*H*. *pylori* eradication.

**Table 4:** Comparison of Relative Abundance of Genera of the Gut Microbiome at Baseline and 12 Months-Post *H*. *pylori* Eradication.

	Mean relative abundance		Comparison of relative abundance at
Genus (species)	(95% CI*)		baseline and 12 months post-eradication
	Baseline	12 Months-Post Eradication	(p-value)^a^
*Dialister*	0.20%	0.71%	0.033
	(0.06%-0.38%)	(0.33%-1.13%)	
*Agrobacterium*	4.34E-4%	0%	0.031
	(1.40E-4%-7.81E-4%)		
*Helicobacter*	0%	2.17E-4%	0.041
		(4.10E-5%-3.94E-4%)	
(*H*. *pylori*)	0%	2.17E-4%	0.041
		(5.80E-5%-4.05E-4%)	

*Bootstrapped 95% confidence interval (CI) was based on 1000 replicates.

^a^Paired-Samples T Test was used in comparing baseline and post-*H*. *pylori* eradication.

**Table 5:** Comparison of Relative Abundance of Genera of the Gut Microbiome at Baseline and 18 Months-Post *H*. *pylori* Eradication.

	Mean relative abundance		Comparison of relative abundance at
Genus (species)	(95% CI*)		baseline and 18 months post-eradication
	Baseline	18 Months-Post Eradication	(p-value)^a^
*Helicobacter*	0%	5.90E-4%	0.033
		(2.37E-4%-9.27E-4%)	
(*H*. *pylori*)	0%	5.86E-4%	0.033
		(2.05E-4%-9.04E-4%)	
Unknown	2.02E-4%	1.28E-3	0.036
(Phylum: *TM7*;	(0%-4.3E-4%)	(6.13E-4%-1.86E-3%)	
Class: *TM7-3*;			
Order: *I025*;			
Family: *Rs-045*)			

*Bootstrapped 95% confidence interval (CI) was based on 1000 replicates.

^a^Paired-Samples T Test was used in comparing baseline and post-*H*. *pylori* eradication.

To examine for the presence of enterohepatic *Helicobacter* species (EHS), we used the generated OTU table to further summarize microbiome communities up to species level. The generated 16S rRNA data showed that the only *Helicobacter* species detected was *H*. *pylori* (Table 4, Table 5 and S1 File)

## Discussion

To our knowledge, the effect of *H*. *pylori* eradication on the gut microbiome has yet to be investigated in *H*. *pylori*-positive healthy young Malaysian adult. *H*. *pylori*-positive volunteers were given eradication therapy and the same cohort of volunteers was subsequently followed up for 6, 12, and 18 months post-eradication. We performed diversity analysis to study the effect of *H*. *pylori* eradication on the gut microbial communities. The lack of significance within each time-point group as well as between the different time-point groups demonstrated that the microbial diversity of the gut microbiome of the volunteers was equally rich and proportional. In addition, following the eradication of *H*. *pylori*, the bacterial communities were not affected. The bacterial communities were similar pre- and post-*H*. *pylori* eradication. Therefore, these results suggested that eradication of *H*. *pylori* may not remarkably interrupt the composition and structure of the gut microbiome.

Irrespective of the *H*. *pylori* eradication status, the general profile of the gut microbiome of the volunteers in our ESSAY study was in accordance with previous findings. As reported elsewhere, most bacterial species in the human and mouse gut was dominated by phyla *Bacteroidetes* and *Firmicutes* [43]. Less abundant bacteria phyla such as *Actinobacteria*, *Proteobacteria*, *Verrucromicrobia*, and as well as *Euryarcheaota* (mainly methanogenic archae *Methaobrevibacter smithii*) were also present [44, 45]. It has been reported that the general profile of the bacteria community of an individual at different body habitats seems to be reasonably stable over time [46].

Despite that the general profile of the gut microbiome was similar pre- and post-*H*. *pylori* eradication, our metagenomics study revealed some changes in the bacterial communities at the phylum and genus levels that are notable. Twelve months post-*H*. *pylori* eradication, the relative abundance of *Bacteroidetes* in the stool microbiome of the volunteers decreased 15% with relative increase in *Firmicutes*, as compared to stool microbiome at Baseline. There are growing evidences that indicated the inverse relationship between *H*. *pylori* prevalence and rate of overweight/obesity. Hence, the gradual decrease of the *H*. *pylori* colonization that has been observed in recent decades could be causally related to the human epidemic obesity [47]. Studies in human and mice have shown that obesity is associated with changes in the composition of the gut microbiome. An early study reported that genetically obese ob/ob mice had a 50% reduction in the abundance of *Bacteroidetes* and proportional increase in *Firmicutes* [43]. Study in human also demonstrated enrichment in *Firmicutes* and a corresponding reduction in *Bacteroidetes* levels in the microbiota of obese individuals; after weight loss, the *Bacteroidetes*-to-*Firmicutes* ratio normalized to the level observed in lean individuals [48].*Bacteroidetes* and *Firmicutes* have been associated with the regulation of lipid and bile acid metabolism as well as energy homeostasis in host [49, 50]. Essentially, it has been demonstrated that perturbations of bile acid-mediated signaling pathway influence risk of metabolic complications such as obesity and diabetes [51]. Eighteen months post-eradication, however, the relative abundance of *Bacteroidetes* and *Firmicutes* seems to be restoring to the Baseline levels with the enrichment of *Proteobacteria*.

In a recent study that investigated the short- and long-term effects of clarithromycin and metronidazole treatment, a dramatic decline in *Actinobacteria* in both throat and feces was reported immediately after *H*. *pylori* eradication therapy. Although the diversity of the microbiome subsequently recovered to resemble the pre-treatment states, the microbiota remained perturbed in some cases for up to four years post-treatment [52]. Correspondingly, in our study, the relative abundance of phylum *Actinobacteria* decreased 6 months-post eradication, and at 12 and 18 months post-eradication, it had increased to resemble to or higher than the Baseline level. This result indicated that broad-spectrum antibiotics used in *H*. *pylori* eradication treatment are also capable of inhibiting a range of Gram-positive and Gram-negative bacteria as well as other bacteria besides eradicatin*g H*. *pylori* [53, 54]. It was reported that high-level colonization of the human gut by *Verrucomicrobia* following broad-spectrum antibiotic treatment [55]. Thus, the increase of phylum *Verrucomicrobia* 6 months post-eradication could be also attributed to the broad-spectrum antibiotic treatment used in *H*. *pylori* eradication therapy. However, at 12 and 18 months post-eradication, it seems to be restoring to the Baseline level.

Another interesting finding was observed for phylum *Proteobacteria*. The relative abundance of *Proteobacteria* decreased 6 months post-*H*. *pylori* eradication but then it increased to even higher than the Baseline state at 12 and 18 months post-eradication. This finding may correlate with our observation at the genus level of the gut microbiome where *Helicobacter* was found 12 and 18 months post-eradication but not during Baseline and 6 months post-eradication. It has been reported that besides *H*. *pylori*, EHS can also colonize the mucosal surfaces of the intestinal tract and/or the liver of humans, mammals and birds [56]. Our study showed that the *Helicobacter* species detected in the stool samples at 12 and 18 months post-eradication was not EHS but *H*. *pylori*. The sole identification of *H*. *pylori* is not due to the lack of taxonomic representation for *Helicobacter* species in Greengenes database [36]. A study published recently showed that 16S rRNA gene can be used to differentiate between gastric *Helicobacter* and EHS although it is not sufficient to distinguish between different EHS [57].

Before the *H*. *pylori*-positive volunteers were given the eradication regimen, *H*. *pylori* was still attached to the gastric mucosa of the stomach and therefore, it may be the reason why it was not detected in their stool samples. Although *H*. *pylori* is generally viewed as a non-invasive pathogen, some *in vivo* and *in vitro* studies have demonstrated otherwise. *H*. *pylori* was found to reside in the vacuole in the cytoplasm, replicate on the cell membrane to form a microcolony, multiply in macrophages and bone marrow-derived dendritic cells, replicate in epithelial cells, and repopulate the extracellular space after the extracellular bacterial population has been killed by gentamicin for up to 3 days [58–63]. Chu et al. also reported that some coccoid forms of *H*. *pylori* were present on the plasma membrane of epithelial cells 18 hours post-*H*. *pylori* infection [63]. All of these studies showed that *H*. *pylori* may be a facultative intracellular organism [64, 65]. When the *H*. *pylori*-positive volunteers were given *H*. *pylori* eradication therapy, most of the *H*. *pylori* colonized on the gastric mucosa may be killed, but some of them may have invaded the gastric epithelial cells and/or antigen-presenting cells and turned into non-culturable but viable, metabolizing coccoid forms under the stress of antibiotics. The dormant coccoid form is resistant to antibiotic and can spread to infect other cells in the absence of an effective concentration of antibiotic [63]. In addition, Tan et al. recently published a report suggesting that even at low to moderate multiplicity of infection (MOI 10), *H*. *pylori* may impede the proliferation of macrophages by disrupting the cell cycle-associated genes and such disruption may be an immunoevasive strategy utilized by *H*. *pylori* [66]. It is likely that *H*. *pylori* utilizes the advantage of ecological niche to replicate intracellularly and survive the antibacterial therapy. This may explain the transient disappearance of *H*. *pylori* immediately following *H*. *pylori* eradication therapy but reappeared at 12–18 months later.

To further strengthen our findings, we performed proteomics analysis on these stool samples using LC-MS approach. Consistent with the detection of *H*. *pylori 16S rRNA* in these stool samples, *H*. *pylori* proteins were also detected (S1 Table) confirming the presence of the bacterium and their viability. In addition, we also managed to call back the volunteers (whom stool samples were detected with *Helicobacter* 16S rRNA) for UBT at 18 months post-*H*.*pylori* eradication. However, all of them were found to be negative for *H*. *pylori* by UBT (data not shown). Negative UBT results at 18 months post-*H*. *pylori* eradication ruled out the possibility of recrudescence or reinfection of *H*. *pylori* in the volunteers. A previous report showed that coccoid forms of *H*. *pylori* may give false negative result for UBT [67] as they produced low level of urease as compared to spiral forms [68, 69]. These may explain the negative UBT results of these volunteers despite detection of *H*. *pylori* DNA and proteins in their stool samples. Our finding has also shown that there is a possibility of *H*. *pylori* can be shed through feces and supported the notion that *H*. *pylori* may be transmitted through fecal-oral route via contaminated water or food [70].

The genus *Anaerofustis* was proposed and classified as *A*. *stercorihominis* sp. nov. under phylum *Firmicutes* and class *Clostridia* to accommodate a phylogenetically distinct Gram-positive, strictly anaerobic, catalase-negative, rod-shaped organism isolated from human feces. It was found to produce acetate and butyrate as end products of glucose fermentation [71]. The significant increment of the relative abundance of *Anaerofustis* 6 months-post eradication could be attributed to the anti-inflammatory and antimicrobial properties possessed by butyrate-producing bacteria [72] that may play a role in restoring the delicate balance between human host and the perturbed gut microbiome. Butyrate producing bacteria produce SCFA such as acetate, butyrate, and propionate [19] through fermentation; the presence of SCFA is believed to be associated with reduced inflammation [73] and has an important effect on colonic health [74, 75]. At 12- & 18 months-post eradication, the relative abundance of *Anaerofustis* was returned to Baseline level after human gut microbiome was restored.

Interestingly, the relative abundance of another SCFA-producing bacteria genus, *Phascolarctobacterium*, also showed significant increment 6 months-post eradication. *Phascolarctobacterium* is also a genus of *Firmicutes* bacteria classified within the class of *Clostridia*. *P*. *succinatutens* sp. nov. isolated by Watanabe and co-workers recently from human feces. *P*. *succinatutens* sp. nov. is distributed broadly in the gut as subdominant members that may adapt to the intestinal environment by specializing to utilize the succinate generated by other bacterial species to produce propionate [76], which may also act as a health-promoting microbial metabolite in the human gut [77] post-*H*. *pylori* eradication to aid in the restoration of the perturbed microbiome. On the side note, SCFA was also reported to stimulate the release of hormone PYY and GLP-1 from rodent enteroendocrine L cells via activation of the G-protein-coupled free fatty acid receptor (FFAR) 2 [78–80]. Of the SCFA produced by colonic fermentation of dietary fibre, propionate has the highest affinity for FFAR 2 [81, 82]. Recently, the first-in-human study also demonstrated that direct delivery of propionate to the colon acutely increases the release of PYY and GLP-1 [83]. In a study reported recently by our group has also shown that *H*. *pylori* eradication was associated with long term elevation of active amylin, PYY, and GLP-1 in the serum [29]. By this mechanism of SCFA-linked G-protein-coupled receptor activation, the gut microbiota may contribute markedly to increased nutrient uptake and deposition, contributing to the development of metabolic disorders [84]. Hence, there is a possibility that eradication of *H*. *pylori* may cause dybiosis which in turn influence the human energy metabolism and lead to the development of obesity.

The genus *Ruminococcus* belongs to phylum *Firmicutes* and corresponds to 5–15% of the total bacterial population in the colon [85, 86]. Currently, the genus *Ruminococcus* is divided into two phylogenetically separate groups which are categorized under two separate families *Ruminococcaceae* [87] and *Lachnospiraceae* [88] with numerous misclassified *Ruminococcus* species [89]. Thus, although the relative abundance of this genus was found to have increased significantly 6 months-post eradication, we could not decipher the effect of *H*. *pylori* eradication on this bacteria genus. Similarly, genus *TM7* is a recently described candidate division of the domain Bacteria, which is currently known only from environmental 16S ribosomal DNA sequence data [90]. Candidate division TM7 is found in a diverse range of environment habitats [90–93] and human body sites [93–97]. These microorganisms have been suggested to play an important role in the early stages of inflammatory mucosal processes, probably by modifying growth conditions for competing bacterial populations [93, 94]. However, we could not elucidate the effect of H. pylori eradication on these organisms in relation to health diseases because they have been uncultivable, with no pure-culture representatives.

In addition to *Helicobacter*, genera *Dialister* and *Agrobacterium* were also found to have changed significantly 12 months post-*H*. *pylori* eradication. Although the clinical significance of *Dialister* spp. and *Agrobacterium* associated with any disease or infection following *H*. *pylori* eradication remains unknown for the time being, it is noteworthy that the significant changes of the relative abundance of these genera 12 months-post eradication.

Our preliminary stool metagenomics study has shown that the eradication of *H*. *pylori* caused perturbation of the gut microbiome and may indirectly affect the health of human. Clinicians should be aware of the effect of broad spectrum antibiotics used in *H*.*pylori* eradication regime and be more cautious in the clinical management of *H*. *pylori* infection, particularly patients from the immunocompromised group. Nonetheless, high throughput experimental approaches such as whole genome shotgun sequencing and metatranscriptomics with bigger sample size is required to verify the observation of this study and also to reveal the complex gene repertoire of the gut microbiome and consequences of *H*. *pylori* eradication in modulating human health.

## Supporting Information

S1 Fig Unweighted Principal Coordinate Analysis (PCoA) plots. Unweighted PCoA plots generated in beta diversity analysis for **A.** Baseline vs. 6 months-post eradication, **B.** Baseline vs. 12 months-post eradication, and **C. B**aseline vs. 18 months-post eradication.
(PDF)

S2 Fig Weighted Principal Coordinate Analysis (PCoA) plots. Weighted PCoA plots generated in beta diversity analysis for **A.** Baseline vs. 6 months-post eradication, **B.** Baseline vs. 12 months-post eradication, and **C. B**aseline vs. 18 months-post eradication.
(PDF)

S1 File Relative abundance of species of the gut microbiome at Baseline, 6, 12 and 18 Months-Post *H*. *pylori* eradication. (XLSX)

S1 Table List of proteins identified in the four stool samples of 12 months post-*H*. *pylori* eradication. (PDF)

## References

[1] Savage DC. Microbial ecology of the gastrointestinal tract. Annu Rev Microbiol. 1977;31:107–133. 334036 10.1146/annurev.mi.31.100177.000543

[2] Ley RE, Hamady M, Lozupone C, Turnbaugh PJ, Ramey RR, Bircher JS, et al Evolution of mammals and their gut microbes. Science. 2008;320(5883):1647–1651. 10.1126/science.1155725 18497261 PMC2649005

[3] Hooper LV, Midtvedt T, Gordon JI. How host-microbial interactions shape the nutrient environment of the mammalian intestine. Annu Rev Nutr. 2002;22:283–307. 12055347 10.1146/annurev.nutr.22.011602.092259

[4] O'Hara AM, Shanahan F. The gut flora as a forgotten organ. EMBO Rep. 2006;7(7):688–693. 16819463 10.1038/sj.embor.7400731 PMC1500832

[5] Hooper LV, Wong MH, Thelin A, Hansson L, Falk PG, Gordon JI. Molecular analysis of commensal host-microbial relationships in the intestine. Science. 2001;291(5505):881–884. 11157169 10.1126/science.291.5505.881

[6] Rawls JF, Mahowald MA, Ley RE, Gordon JI. Reciprocal gut microbiota transplants from zebrafish and mice to germ-free recipients reveal host habitat selection. Cell. 2006;127(2):423–433. 17055441 10.1016/j.cell.2006.08.043 PMC4839475

[7] Li M, Wang B, Zhang M, Rantalainen M, Wang S, Zhou H, et al Symbiotic gut microbes modulate human metabolic phenotypes. Proc Natl Acad Sci U S A. 2008;105(6):2117–2122. 10.1073/pnas.0712038105 18252821 PMC2538887

[8] Cantarel BL, Lombard V, Henrissat B. Complex carbohydrate utilization by the healthy human microbiome. PLoS One. 2012;7(6):e28742 10.1371/journal.pone.0028742 22719820 PMC3374616

[9] Cani PD, Delzenne NM. The role of the gut microbiota in energy metabolism and metabolic disease. Curr Pharm Des. 2009;15(13):1546–1558. 19442172 10.2174/138161209788168164

[10] Harris K, Kassis A, Major G, Chou CJ. Is the gut microbiota a new factor contributing to obesity and its metabolic disorders? J Obes. 2012;2012:14 10.1155/2012/879151 PMC3270440 22315672

[11] Tilg H, Kaser A. Gut microbiome, obesity, and metabolic dysfunction. J Clinc Invest. 2011;121(6):2126–2132. 10.1172/JCI58109 PMC3104783 21633181

[12] Cani PD, Delzenne NM. Gut microflora as a target for energy and metabolic homeostasis. Curr Opin Clin Nutr Metab Care. 2007;10(6):729–734. 18089955 10.1097/MCO.0b013e3282efdebb

[13] Sekirov I, Russell SL, Antunes LCM, Finlay BB. Gut microbiota in health and disease. Physiol Rev. 2010;90(3):859–904. 10.1152/physrev.00045.2009 20664075

[14] Endt K, Stecher B, Chaffron S, Slack E, Tchitchek N, Benecke A, et al The microbiota mediates pathogen clearance from the gut lumen after non-typhoidal Salmonella diarrhea. PLoS Pathog. 2010;6(9):e1001097 10.1371/journal.ppat.1001097 20844578 PMC2936549

[15] Buffie CG, Pamer EG. Microbiota-mediated colonization resistance against intestinal pathogens. Nat Rev Immunol. 2013;13(11):790–801. 10.1038/nri3535 24096337 PMC4194195

[16] Kelly D, Conway S, Aminov R. Commensal gut bacteria: mechanisms of immune modulation. Trends Immunol. 2005;26(6):326–333. 15922949 10.1016/j.it.2005.04.008

[17] DiBaise JK, Frank DN, Mathur R. Impact of the gut microbiota on the development of obesity: current concepts. Am J Gastroenterol Suppl. 2012;1(1):22–27.

[18] Tremaroli V, Backhed F. Functional interactions between the gut microbiota and host metabolism. Nature. 2012;489(7415):242–249. 10.1038/nature11552 22972297

[19] Sun J, Chang EB. Exploring gut microbes in human health and disease: Pushing the envelope. Genes Dis. 2014;1(2):132–139. 25642449 10.1016/j.gendis.2014.08.001 PMC4310008

[20] Clyne M, Dolan B, Reeves EP. Bacterial factors that mediate colonization of the stomach and virulence of *Helicobacter pylori*. FEMS Microbiol Lett. 2007;268(2):135–143. 17313591 10.1111/j.1574-6968.2007.00648.x

[21] Kusters JG, van Vliet AH, Kuipers EJ. Pathogenesis of *Helicobacter pylori* infection. Clin Microbiol Rev. 2006;19(3):449–490. 16847081 10.1128/CMR.00054-05 PMC1539101

[22] Linz B, Balloux F, Moodley Y, Manica A, Liu H, Roumagnac P, et al An African origin for the intimate association between humans and *Helicobacter pylori*. Nature. 2007;445(7130):915–918. 17287725 10.1038/nature05562 PMC1847463

[23] Francois F, Roper J, Joseph N, Pei Z, Chhada A, Shak JR, et al The effect of *H*. *pylori* eradication on meal-associated changes in plasma ghrelin and leptin. BMC Gastroenterol. 2011;11:37. doi: 1471-230X-11-37 [pii] 10.1186/1471-230X-11-37 21489301 PMC3089783

[24] Nwokolo CU, Freshwater DA, O'Hare P, Randeva HS. Plasma ghrelin following cure of *Helicobacter pylori*. Gut. 2003;52(5):637–640. 12692045 10.1136/gut.52.5.637 PMC1773634

[25] Osawa H. Ghrelin and *Helicobacter pylori* infection. World J Gastroenterol. 2008;14(41):6327–6333. 19009647 10.3748/wjg.14.6327 PMC2766113

[26] Chen Y, Blaser MJ. *Helicobacter pylori* colonization is inversely associated with childhood asthma. J Infect Dis. 2008;198(4):553–560. 10.1086/590158 18598192 PMC3902975

[27] Amberbir A, Medhin G, Erku W, Alem A, Simms R, Robinson K, et al Effects of *Helicobacter pylori*, geohelminth infection and selected commensal bacteria on the risk of allergic disease and sensitization in 3-year-old Ethiopian children. Clin Exp Allergy. 2011;41(10):1422–1430. 10.1111/j.1365-2222.2011.03831.x 21831135

[28] Amnon S, Robert MG. *Helicobacter pylori* is a risk factor for colonic neoplasms. Am J Gastroenterol. 2012;108(2):208–215. 10.1038/ajg.2012.407 23208272

[29] Yap TW-C, Leow AH-R, Azmi AN, Francois F, Perez-Perez GI, Blaser MJ, et al Changes in metabolic hormones in Malaysian young adults following *Helicobacter pylori* eradication. PLoS One. 2015;10(8):e0135771 10.1371/journal.pone.0135771 26291794 PMC4546342

[30] Peterson J, Garges S, Giovanni M, McInnes P, Wang L, Schloss JA, et al The NIH Human Microbiome Project. Genome Res. 2009;19(12):2317–2323. 10.1101/gr.096651.109 19819907 PMC2792171

[31] Klindworth A, Pruesse E, Schweer T, Peplies J, Quast C, Horn M, et al Evaluation of general 16S ribosomal RNA gene PCR primers for classical and next-generation sequencing-based diversity studies. Nucleic Acids Res. 2013;41(1):e1 10.1093/nar/gks808 22933715 PMC3592464

[32] Bartram AK, Lynch MD, Stearns JC, Moreno-Hagelsieb G, Neufeld JD. Generation of multimillion-sequence 16S rRNA gene libraries from complex microbial communities by assembling paired-end illumina reads. Appl Environ Microbiol2011;77(11):3846–3852. 10.1128/AEM.02772-10 PMC3127616 21460107

[33] Available: http://hannonlab.cshl.edu/fastx_toolkit/.

[34] Zhang J, Kobert K, Flouri T, Stamatakis A. PEAR: a fast and accurate Illumina Paired-End reAd mergeR. Bioinformatics. 2014;30(5):614–620. 10.1093/bioinformatics/btt593 24142950 PMC3933873

[35] Caporaso JG, Kuczynski J, Stombaugh J, Bittinger K, Bushman FD, Costello EK, et al QIIME allows analysis of high-throughput community sequencing data. Nature Methods. 2010;7(5):335–336. 10.1038/nmeth.f.303 20383131 PMC3156573

[36] McDonald D, Price MN, Goodrich J, Nawrocki EP, DeSantis TZ, Probst A, et al An improved Greengenes taxonomy with explicit ranks for ecological and evolutionary analyses of bacteria and archaea. ISME J. 2012;6(3):610–618. 10.1038/ismej.2011.139 22134646 PMC3280142

[37] Edgar RC. Search and clustering orders of magnitude faster than BLAST. Bioinformatics. 2010;26(19):2460–2461. 10.1093/bioinformatics/btq461 20709691

[38] Caporaso JG, Bittinger K, Bushman FD, DeSantis TZ, Andersen GL, Knight R. PyNAST: a flexible tool for aligning sequences to a template alignment. Bioinformatics. 2010;26(2):266–267. 10.1093/bioinformatics/btp636 19914921 PMC2804299

[39] DeSantis TZ, Hugenholtz P, Larsen N, Rojas M, Brodie EL, Keller K, et al Greengenes, a chimera-checked 16S rRNA gene database and workbench compatible with ARB. Appl Environ Microbiol. 2006;72(7):5069–5072. 16820507 10.1128/AEM.03006-05 PMC1489311

[40] Price MN, Dehal PS, Arkin AP. FastTree 2—approximately maximum-likelihood trees for large alignments. PLoS One. 2010;5(3):e9490 10.1371/journal.pone.0009490 20224823 PMC2835736

[41] Lozupone C, Knight R. UniFrac: a New Phylogenetic method for comparing microbial communities. Appl Environ Microbiol. 2005;71(12):8228–8235. 16332807 10.1128/AEM.71.12.8228-8235.2005 PMC1317376

[42] Vazquez-Baeza Y, Pirrung M, Gonzalez A, Knight R. EMPeror: a tool for visualizing high-throughput microbial community data. GigaScience. 2013;2(1):16 10.1186/2047-217X-2-16 24280061 PMC4076506

[43] Ley RE, Bäckhed F, Turnbaugh P, Lozupone CA, Knight RD, Gordon JI. Obesity alters gut microbial ecology. Proc Natl Acad Sci U S A. 2005;102(31):11070–11075. 16033867 10.1073/pnas.0504978102 PMC1176910

[44] Eckburg PB, Bik EM, Bernstein CN, Purdom E, Dethlefsen L, Sargent M, et al Diversity of the human intestinal microbial flora. Science. 2005;308(5728):1635–1638. 15831718 10.1126/science.1110591 PMC1395357

[45] Qin J, Li R, Raes J, Arumugam M, Burgdorf KS, Manichanh C, et al A human gut microbial gene catalogue established by metagenomic sequencing. Nature. 2010;464(7285):59–65. 10.1038/nature08821 20203603 PMC3779803

[46] Costello EK, Lauber CL, Hamady M, Fierer N, Gordon JI, Knight R. Bacterial community variation in human body habitats across space and time. Science. 2009;326(5960):1694–1697. 10.1126/science.1177486 19892944 PMC3602444

[47] Lender N, Talley NJ, Enck P, Haag S, Zipfel S, Morrison M, et al Associations between *Helicobacter pylori* and obesity—an ecological study. Aliment Pharmacol Ther. 2014;40(1):24–31. 10.1111/apt.12790 24832176

[48] Ley RE, Turnbaugh PJ, Klein S, Gordon JI. Microbial ecology: human gut microbes associated with obesity. Nature. 2006;444(7122):1022–1023. 17183309 10.1038/4441022a

[49] Turnbaugh PJ, Ley RE, Mahowald MA, Magrini V, Mardis ER, Gordon JI. An obesity-associated gut microbiome with increased capacity for energy harvest. Nature. 2006;444(7122):1027–1131. 17183312 10.1038/nature05414

[50] Van Eldere J, Celis P, De Pauw G, Lesaffre E, Eyssen H. Tauroconjugation of cholic acid stimulates 7 alpha-dehydroxylation by fecal bacteria. Appl Environ Microbiol. 1996;62(2):656–661. 8593067 10.1128/aem.62.2.656-661.1996 PMC167832

[51] Martin FP, Dumas ME, Wang Y, Legido-Quigley C, Yap IK, Tang H, et al A top-down systems biology view of microbiome-mammalian metabolic interactions in a mouse model. Mol Syst Biol. 2007;3:112 17515922 10.1038/msb4100153 PMC2673711

[52] Jakobsson HE, Jernberg C, Andersson AF, Sjolund-Karlsson M, Jansson JK, Engstrand L. Short-term antibiotic treatment has differing long-term impacts on the human throat and gut microbiome. PLoS One. 2010;5(3):e9836 10.1371/journal.pone.0009836 20352091 PMC2844414

[53] Peters DH, Clissold SP. Clarithromycin. A review of its antimicrobial activity, pharmacokinetic properties and therapeutic potential. Drugs. 1992;44(1):117–164. 1379907 10.2165/00003495-199244010-00009

[54] Elliott TSJ, Stone JW. Review article: metronidazole and the anaerobic gut flora. Aliment Pharmacol Ther. 1990;4(3):227–238. 2104087 10.1111/j.1365-2036.1990.tb00467.x

[55] Dubourg G, Lagier JC, Armougom F, Robert C, Audoly G, Papazian L, et al High-level colonisation of the human gut by Verrucomicrobia following broad-spectrum antibiotic treatment. Int J Antimicrob Agents. 2013;41(2):149–155. 10.1016/j.ijantimicag.2012.10.012 23294932

[56] Schauer DB. Enterohepatic Helicobacter Species In: Mobley HLT, Mendz GL, Hazell SL, editors. *Helicobacter pylori*: Physiology and Genetics. Washington DC: ASM Press; 2001.

[57] Ménard A, Buissonnière A, Prouzet-Mauléon V, Sifré E, Mégraud F. The *GyrA* encoded gene: a pertinent marker for the phylogenetic revision of *Helicobacter* genus. Syst Appl Microbiol. 2016. 10.1016/j.syapm.2015.09.008 26829999

[58] Kwok T, Backert S, Schwarz H, Berger J, Meyer TF. Specific entry of *Helicobacter pylori* into cultured gastric epithelial cells via a zipper-like mechanism. Infect Immun. 2002;70(4):2108–2120. 11895977 10.1128/IAI.70.4.2108-2120.2002 PMC127843

[59] Amieva MR, Salama NR, Tompkins LS, Falkow S. *Helicobacter pylori* enter and survive within multivesicular vacuoles of epithelial cells. Cell Microbiol. 2002;4(10):677–690. 12366404 10.1046/j.1462-5822.2002.00222.x

[60] Wang YH, Wu JJ, Lei HY. The autophagic induction in *Helicobacter pylori*-infected macrophage. Exp Biol Med (Maywood). 2009;234(2):171–180. 19064937 10.3181/0808-RM-252

[61] Tan S, Tompkins LS, Amieva MR. *Helicobacter pylori* usurps cell polarity to turn the cell surface into a replicative niche. PLoS Pathog. 2009;5(5):e1000407 10.1371/journal.ppat.1000407 19412339 PMC2669173

[62] Wang YH, Gorvel JP, Chu YT, Wu JJ, Lei HY. *Helicobacter pylori* impairs murine dendritic cell responses to infection. PLoS One. 2010;5(5):e10844 10.1371/journal.pone.0010844 20523725 PMC2877707

[63] Chu YT, Wang YH, Wu JJ, Lei HY. Invasion and multiplication of *Helicobacter pylori* in gastric epithelial cells and implications for antibiotic resistance. Infect Immun. 2010;78(10):4157–4165. 10.1128/IAI.00524-10 20696835 PMC2950335

[64] Dubois A, Boren T. *Helicobacter pylori* is invasive and it may be a facultative intracellular organism. Cell Microbiol. 2007;9(5):1108–1116. 17388791 10.1111/j.1462-5822.2007.00921.x PMC1913845

[65] Petersen AM, Krogfelt KA. *Helicobacter pylori*: an invading microorganism? A review. FEMS Immunol Med Microbiol. 2003;36(3):117–126. 12738380 10.1016/S0928-8244(03)00020-8

[66] Tan GMY, Looi CY, Fernandez KC, Vadivelu J, Loke MF, Wong WF. Suppression of cell division-associated genes by *Helicobacter pylori* attenuates proliferation of RAW264.7 monocytic macrophage cells. Sci Rep. 2015;5 10.1038/srep11046 PMC4468580 26078204

[67] Weingart V, Rüssmann H, Koletzko S, Weingart J, Höchter W, Sackmann M. Sensitivity of a novel stool antigen test for detection of *Helicobacter pylori* in adult outpatients before and after eradication therapy. J Clin Microbiol. 2004;42(3):1319–1321. 15004108 10.1128/JCM.42.3.1319-1321.2004 PMC356865

[68] Nilius M, Strohle A, Bode G, Malfertheiner P. Coccoid like forms (CLF) of *Helicobacter pylori*. Enzyme activity and antigenicity. Zentralbl Bakteriol. 1993;280(1–2):259–272. 8280950 10.1016/s0934-8840(11)80964-3

[69] Hua J, Ho B. Is the coccoid form of *Helicobacter pylori* viable? Microbios. 1996;87(351):103–12. 9032959

[70] Malaty HM. Epidemiology of *Helicobacter pylori* infection In: Sutton P, Mitchell HM, editors. *Helicobacter pylori* in the 21st century. Wallingford: CABI; 2010 pp. 1–12.

[71] Finegold SM, Lawson PA, Vaisanen ML, Molitoris DR, Song Y, Liu C, et al *Anaerofustis stercorihominis* gen. nov., sp. nov., from human feces. Anaerobe. 2004;10(1):41–45. 16701499 10.1016/j.anaerobe.2003.10.002

[72] Hamer HM, Jonkers D, Venema K, Vanhoutvin S, Troost FJ, Brummer RJ. The role of butyrate on colonic function. Aliment Pharmacol Ther. 2008;27(2):104–119. 17973645 10.1111/j.1365-2036.2007.03562.x

[73] Karlsson F, Tremaroli V, Nielsen J, Backhed F. Assessing the human gut microbiota in metabolic diseases. Diabetes. 2013;62(10):3341–3349. 10.2337/db13-0844 24065795 PMC3781439

[74] Szylit O, Andrieux C. Physiological and pathophysiological effects of carbohydrate fermentation. World Rev Nutr Diet. 1993;74:88–122. 8212731 10.1159/000422603

[75] Cummings JH, Macfarlane GT. Colonic microflora: nutrition and health. Nutrition. 1997;13(5):476–478. 9225346 10.1016/s0899-9007(97)00114-7

[76] Watanabe Y, Nagai F, Morotomi M. Characterization of *Phascolarctobacterium succinatutens* sp. nov., an asaccharolytic, succinate-utilizing bacterium isolated from human feces. Appl Environ Microbiol. 2012;78(2):511–518. 10.1128/AEM.06035-11 22081579 PMC3255759

[77] Hosseini E, Grootaert C, Verstraete W, Van de Wiele T. Propionate as a health-promoting microbial metabolite in the human gut. Nutr Rev. 2011;69(5):245–258. 10.1111/j.1753-4887.2011.00388.x 21521227

[78] Tolhurst G, Heffron H, Lam YS, Parker HE, Habib AM, Diakogiannaki E, et al Short-chain fatty acids stimulate glucagon-like peptide-1 secretion via the G-Protein–coupled receptor FFAR2. Diabetes. 2012;61(2):364–371. 10.2337/db11-1019 22190648 PMC3266401

[79] Cherbut C, Ferrier L, Rozé C, Anini Y, Blottière H, Lecannu G, et al Short-chain fatty acids modify colonic motility through nerves and polypeptide YY release in the rat. Am J Physiol. 1998;275(6 Pt 1):G1415–22. 9843779 10.1152/ajpgi.1998.275.6.G1415

[80] Anini Y, Fu-Cheng X, Cuber JC, Kervran A, Chariot J, Rozé C. Comparison of the postprandial release of peptide YY and proglucagon-derived peptides in the rat. Pflügers Arch. 1999;438(3):299–306. 10398859 10.1007/s004240050913

[81] Brown AJ, Goldsworthy SM, Barnes AA, Eilert MM, Tcheang L, Daniels D, et al The orphan G Protein-coupled receptors GPR41 and GPR43 are activated by propionate and other short chain carboxylic acids. J Biol Chem. 2003;278(13):11312–11319. 12496283 10.1074/jbc.M211609200

[82] Le Poul E, Loison C, Struyf S, Springael J-Y, Lannoy V, Decobecq M-E, et al Functional characterization of human receptors for short chain fatty acids and their role in polymorphonuclear cell activation. J Biol Chem. 2003;278(28):25481–25489. 12711604 10.1074/jbc.M301403200

[83] Chambers ES, Viardot A, Psichas A, Morrison DJ, Murphy KG, Zac-Varghese SEK, et al Effects of targeted delivery of propionate to the human colon on appetite regulation, body weight maintenance and adiposity in overweight adults. Gut. 2014 10.1136/gutjnl-2014-307913 PMC4680171 25500202

[84] Erejuwa OO, Sulaiman SA, Ab Wahab MS. Modulation of gut microbiota in the management of metabolic disorders: the prospects and challenges. Int J Mol Sci. 2014;15(3):4158–4188. 10.3390/ijms15034158 24608927 PMC3975390

[85] Chassard C, Scott KP, Marquet P, Martin JC, Del'homme C, Dapoigny M, et al Assessment of metabolic diversity within the intestinal microbiota from healthy humans using combined molecular and cultural approaches. FEMS Microbiol Ecol. 2008;66(3):496–504. 10.1111/j.1574-6941.2008.00595.x 18811647

[86] Ramirez-Farias C, Slezak K, Fuller Z, Duncan A, Holtrop G, Louis P. Effect of inulin on the human gut microbiota: stimulation of *Bifidobacterium adolescentis* and *Faecalibacterium prausnitzii*. British J Nutr. 2009;101(04):541–550. 10.1017/S0007114508019880 18590586

[87] Rainey FA. Family VIII. Ruminococcaceae fam. nov. Bergey’s Manual of Systematic Bacteriology. 2009;3:1016–1043.

[88] Rainey FA. Family V. Lachnospiraceae fam. nov. Bergey's Manual of Systematic Bacteriology. 2009;3:921.

[89] Liu C, Finegold SM, Song Y, Lawson PA. Reclassification of *Clostridium coccoides*, *Ruminococcus hansenii*, *Ruminococcus hydrogenotrophicus*, *Ruminococcus luti*, *Ruminococcus productus* and *Ruminococcus schinkii* as *Blautia coccoides* gen. nov., comb. nov., *Blautia hansenii* comb. nov., *Blautia hydrogenotrophica* comb. nov., *Blautia luti* comb. nov., *Blautia producta* comb. nov., *Blautia schinkii* comb. nov. and description of *Blautia wexlerae* sp. nov., isolated from human faeces. Int J Syst Evol Microbiol. 2008;58(8):1896–1902. 18676476 10.1099/ijs.0.65208-0

[90] Hugenholtz P, Tyson GW, Webb RI, Wagner AM, Blackall LL. Investigation of candidate division TM7, a recently recognized major lineage of the domain bacteria with no known pure-culture representatives. Appl Environ Microbiol. 2001;67(1):411–419. 11133473 10.1128/AEM.67.1.411-419.2001 PMC92593

[91] Ouverney CC, Armitage GC, Relman DA. Single-cell enumeration of an uncultivated TM7 cubgroup in the human subgingival crevice. Appl Environ Microbiol. 2003;69(10):6294–6298. 14532094 10.1128/AEM.69.10.6294-6298.2003 PMC201210

[92] Kumar PS, Griffen AL, Barton JA, Paster BJ, Moeschberger ML, Leys EJ. New bacterial species associated with chronic periodontitis.J Dent Res. 2003;82(5):338–344. 12709498 10.1177/154405910308200503

[93] Brinig MM, Lepp PW, Ouverney CC, Armitage GC, Relman DA. Prevalence of bacteria of division TM7 in human subgingival plaque and their association with disease. Appl Environ Microbiol. 2003;69(3):1687–1694. 12620860 10.1128/AEM.69.3.1687-1694.2003 PMC150096

[94] Kuehbacher T, Rehman A, Lepage P, Hellmig S, Fölsch UR, Schreiber S, et al Intestinal TM7 bacterial phylogenies in active inflammatory bowel disease. J Med Microbiol. 2008;57(12):1569–1576. 19018031 10.1099/jmm.0.47719-0

[95] Dinis JM, Barton DE, Ghadiri J, Surendar D, Reddy K, Velasquez F, et al In search of an uncultured human-associated TM7 bacterium in the environment. PLoS One. 2011;6(6):1–8. 10.1371/journal.pone.0021280 PMC3118805 21701585

[96] Pei Z, Bini EJ, Yang L, Zhou M, Francois F, Blaser MJ. Bacterial biota in the human distal esophagus. Proc Natl Acad Sci U S A. 2004;101(12):4250–4255. 15016918 10.1073/pnas.0306398101 PMC384727

[97] Dewhirst FE, Chen T, Izard J, Paster BJ, Tanner ACR, Yu W-H, et al The human oral microbiome. J Bacteriol. 2010;192(19):5002–5017. 10.1128/JB.00542-10 20656903 PMC2944498

