# Supplementary figures and images for: Helicobacter pylori Eradication Causes Perturbation of the Human Gut Microbiome in Young Adults
Source: PLoS One. 2016 Mar 18;11(3):e0151893. doi: 10.1371/journal.pone.0151893 (PMC4798770; doi:10.1371/journal.pone.0151893)

A

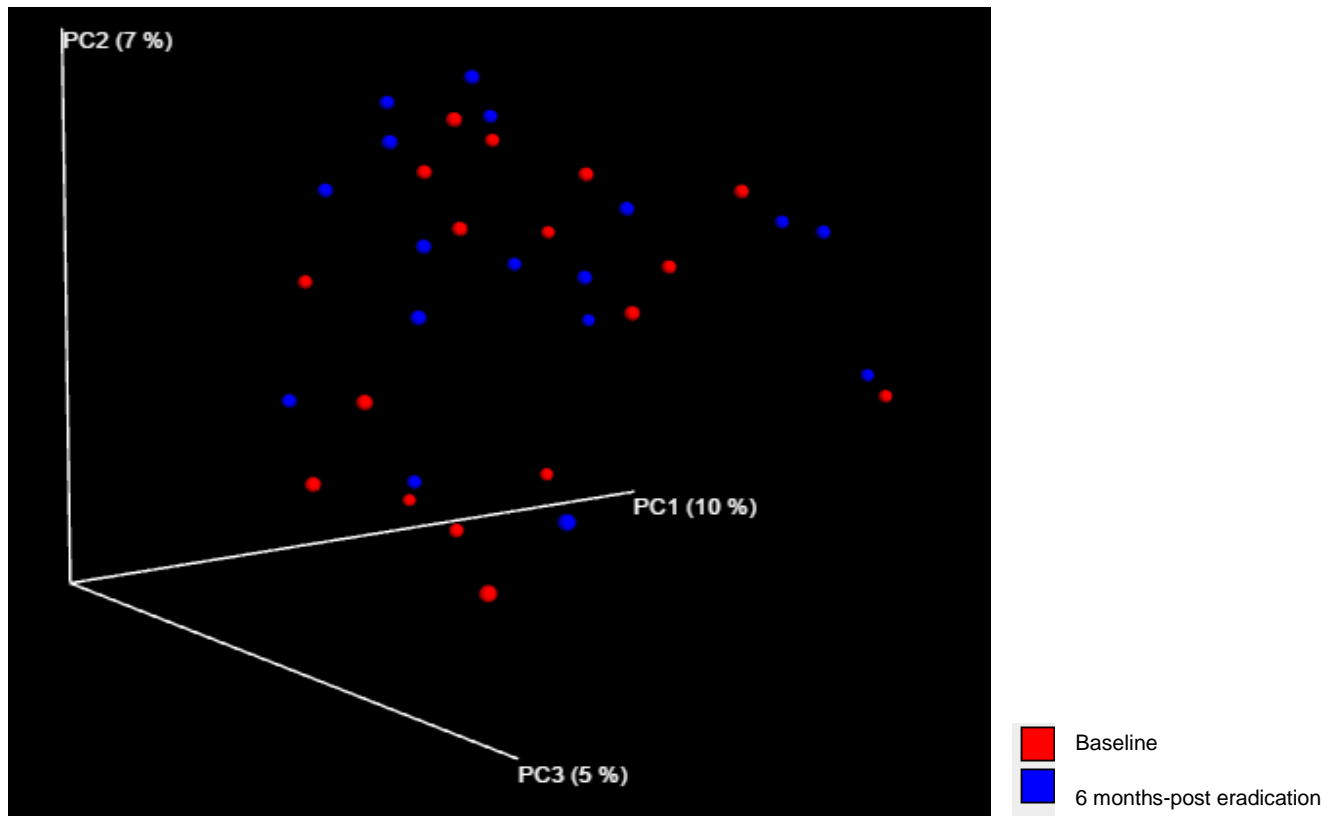

B

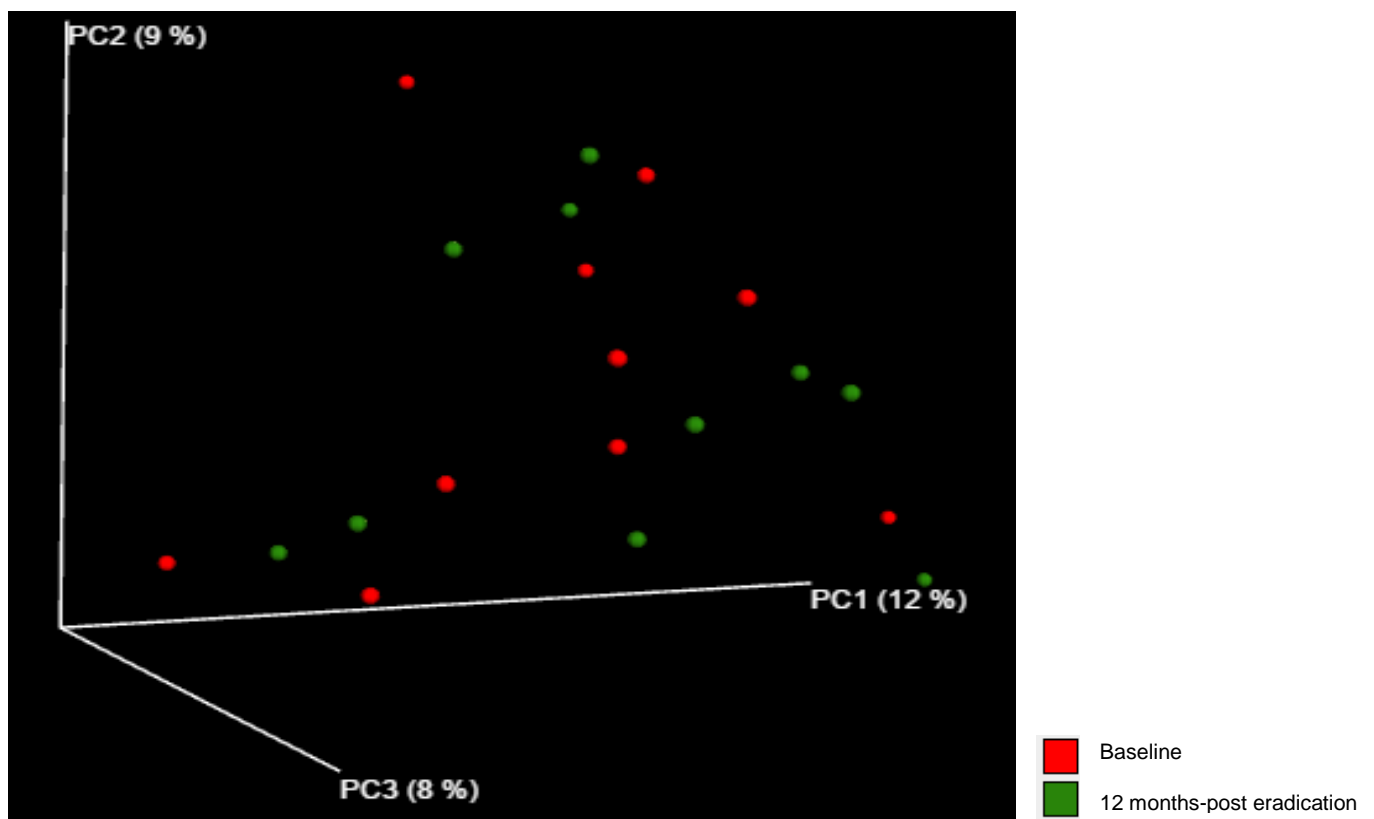

**C**

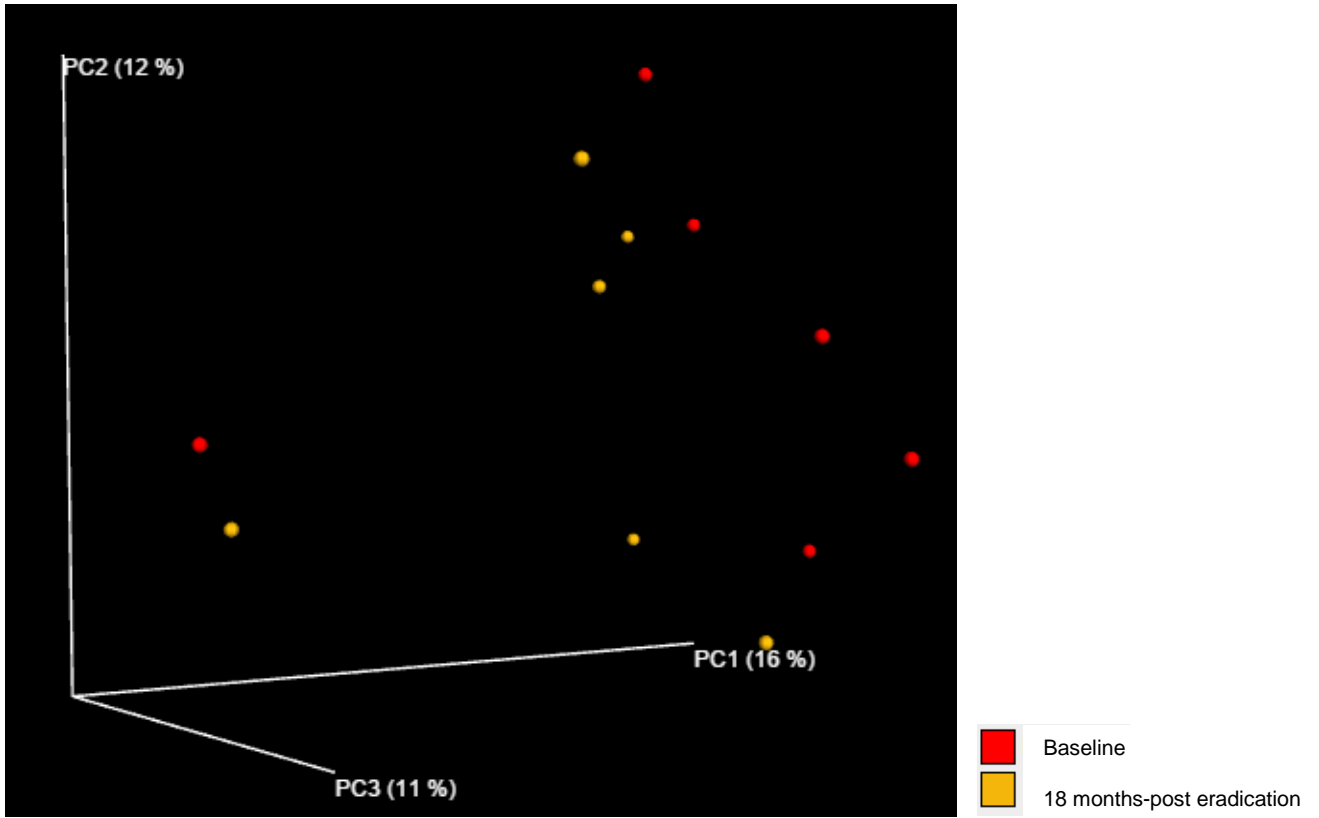

Supplement: S1 Fig — Unweighted PCoA plots generated in beta diversity analysis for A. Baseline vs. 6 months-post eradication, B. Baseline vs. 12 months-post eradication, and C. Baseline vs. 18 months-post eradication. (PDF) [file pone.0151893.s001.pdf]

A

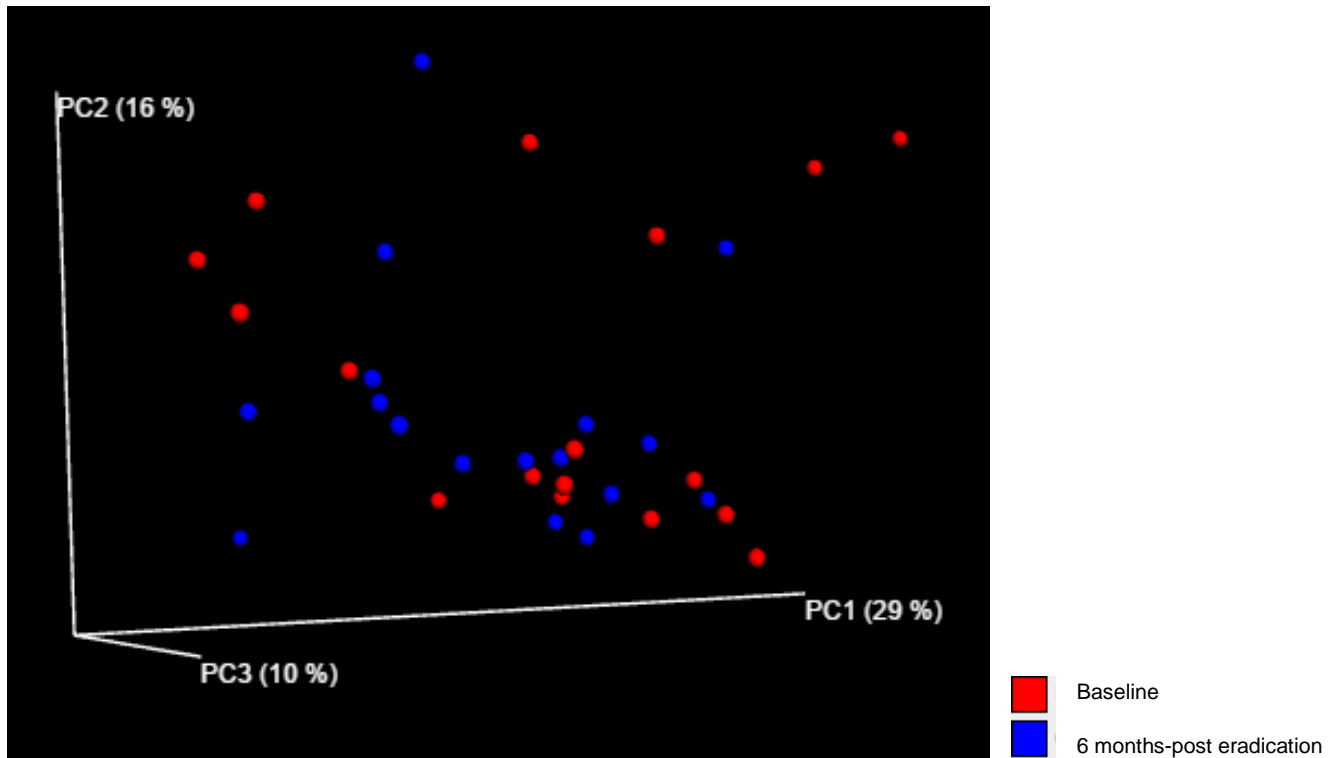

B

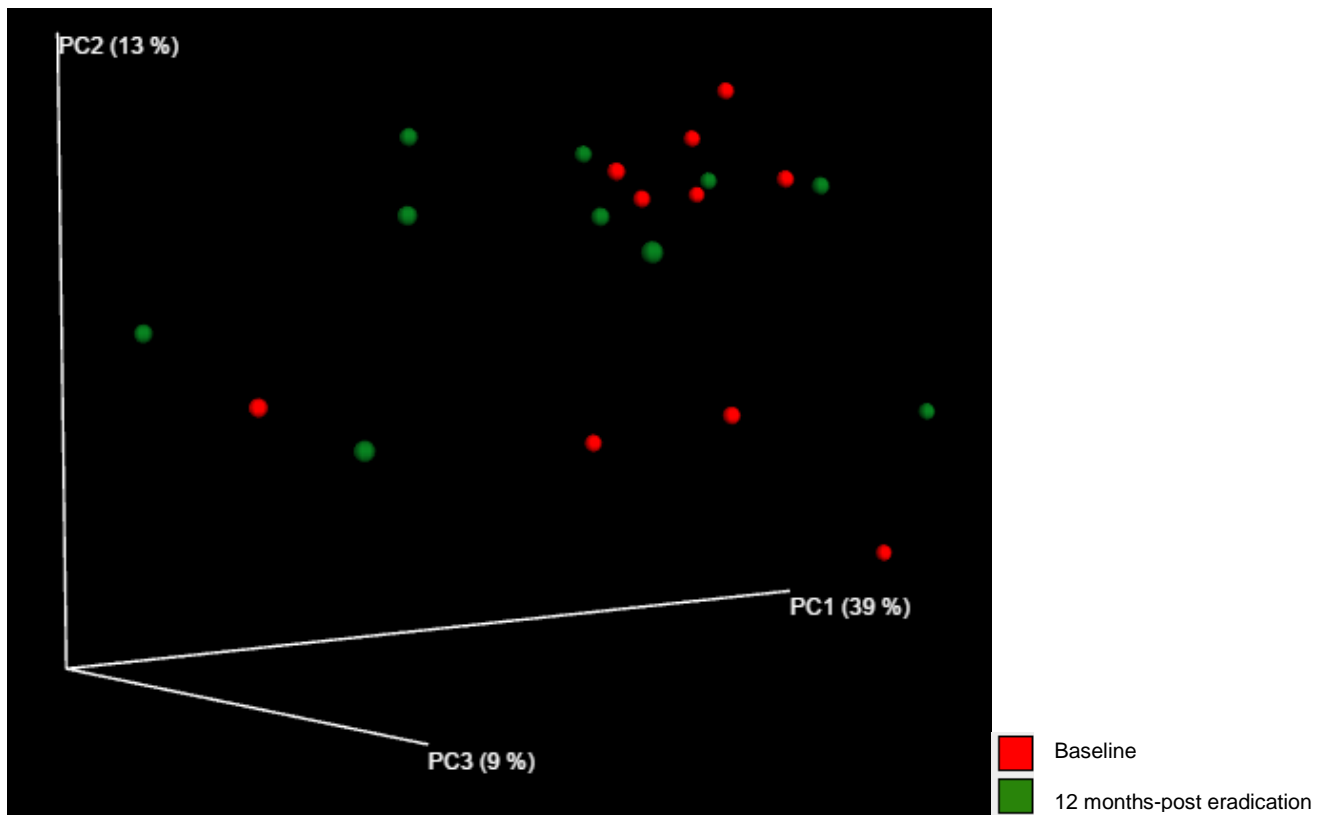

C

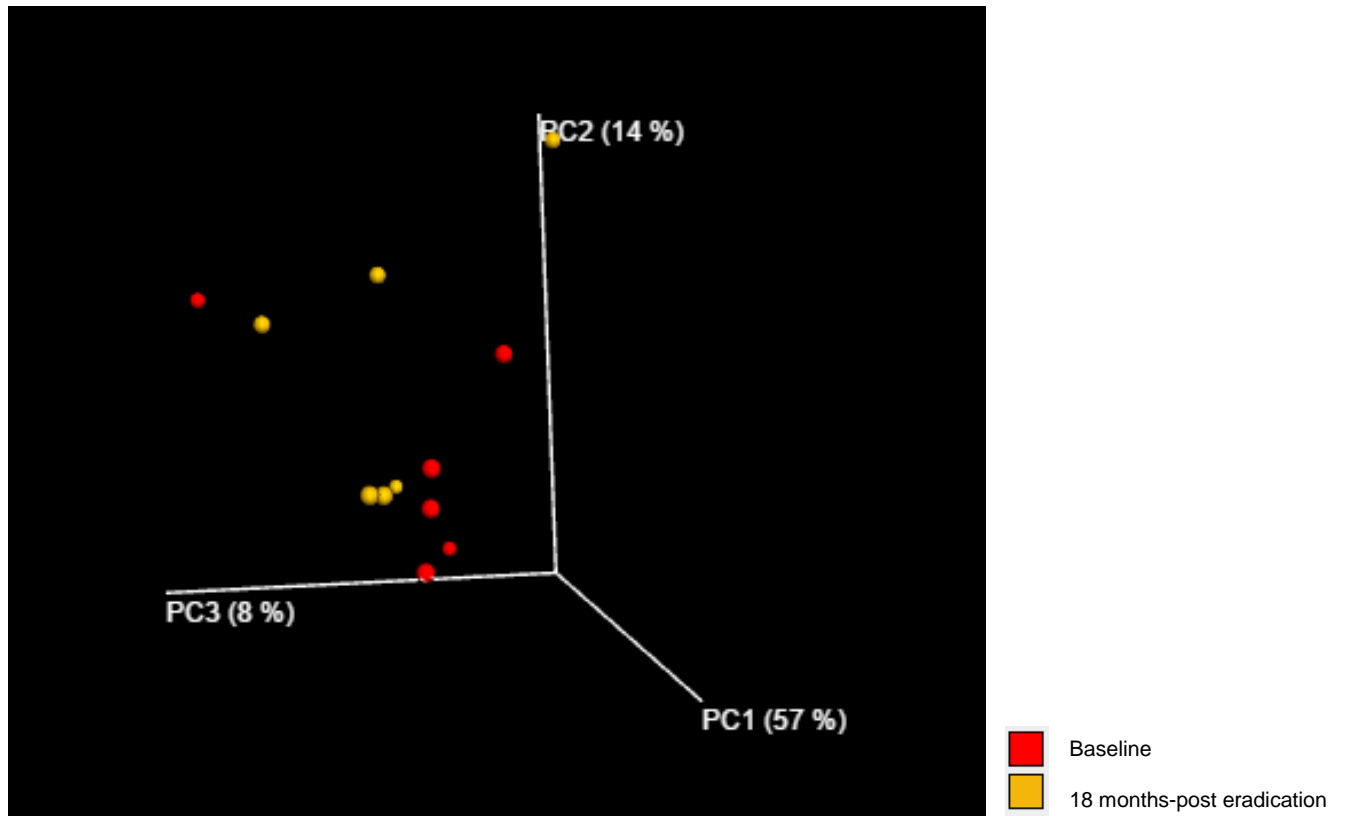

Supplement: S2 Fig — Weighted PCoA plots generated in beta diversity analysis for A. Baseline vs. 6 months-post eradication, B. Baseline vs. 12 months-post eradication, and C. Baseline vs. 18 months-post eradication. (PDF) [file pone.0151893.s002.pdf]
