# Supplementary material for: Helicobacter pylori Eradication Causes Perturbation of the Human Gut Microbiome in Young Adults
Source: PLoS One. 2016 Mar 18;11(3):e0151893. doi: 10.1371/journal.pone.0151893 (PMC4798770; doi:10.1371/journal.pone.0151893)
Supplement: S1 Table — (PDF) [file pone.0151893.s004.pdf]

Helicobacter pylori eradication causes perturbation of the human gut microbiome in young adults

Authors:

Theresa Wan-Chen Yap[1], Han-Ming Gan[2], Alex Hwong-Ruey Leow[3], Ahmad Najib Azmi[3], Fritz Francois[4,5], Guillermo I Perez-Perez[5,6], Martin J Blaser[5,6] , Mun-Fai Loke[1], Khean-Lee Goh[3], *Jamuna Vadivelu[1]

Affiliations:

[1]Department of Medical Microbiology, [3]Department of Medicine, Faculty of Medicine, University of Malaya, 50603 Kuala Lumpur, Malaysia.

[2]School of Science, Monash University Malaysia, 47500 Bandar Sunway, Selangor Darul Ehsan, Malaysia.

[4]New York University Cancer Institute, [5]Department of Medicine, [6]Department of Microbiology, New York University School of Medicine, New York, NY 10016, USA.

Materials and Methods

Protein extraction and Trypsin digestion

Approximately 5 mg of stool samples were lysed and proteins were extracted using the ProteoSpin detergent-free total protein isolation kit (Norgen Biotek, Canada) with the Halt protease and phosphatase inhibitors cocktail (Thermo Scientific, Waltham, MA) included. The lysates were subsequently treated with 10 mM dithiothreitol (DTT; Bio-Rad, Hercules, CA) at 37°C for 10 min and alkylated with 55 mM iodoacetamide (IAA; Bio-Rad) for 30 min at room temperature. The proteins in the sample were digested with1:50 (trypsin: *protein*) of MS-grade Pierce trypsin protease (Thermo Scientific, Waltham, MA) at 37°C overnight. The samples were desalted using a Pierce C-18 spin column (Thermo Scientific, USA) and dried to completeness in a refrigerated CentriVap centrifugal vacuum concentrator (Labconco, Kansas City, MO) before mass spectrometry analysis.

High-Performance Liquid Chromatography Tandem Mass Spetrometry (LC-MS/MS)

Tryptic peptides were analyzed on the 1260 Infinity HPLC-Chip/MS System (Agilent Technologies, Santa Clara, CA). The HPLC-Chip was the Large Capacity C18 Chip (G4240-6210), which comprised a 160 nL enrichment column and a 150 mm analytical column. LCMS-grade water (0.1% formic acid) and acetonitrile (0.1% formic acid) were used as mobile phases A and B respectively. HPLC-grade acetonitrile was from Friendemann Schmidt (Australia), LCMS-grade water from a Milli-Q water

purification system (EMD Millipore, Billerica, MA) and formic acid from Sigma-Aldrich (St.Louis, MO). The nanoflow gradient (%B) used was: 3% at 0 min, 3% at 5 min, 35% at 60 min, 40% at 67 min, 60% at 85 min, 60% at 95 min, 3% at 105 min; stop time: 120 minutes; nanopump flow rate: 0.3 uL/min; capillary pump gradient: 3% B isocratic; capillary pump flow: 2.5 uL/min; chip value position: enrichment at 95 min. An Agilent 6540 Accurate-Mass Q-TOF LC/MS System operated in positive ion mode was used for mass detection, applying the following settings: capillary voltage : 1850 V; drying gas flow: 5 L/min; drying gas temperature: 250°C; fragmenter: 175 V; skimmer: 65 V; acquisition mode: autoMS/MS; scan range: 125-1700 *m/z* (MS), 50-1700 *m/z* (MS/MS); acquisition rate: 15 spectra/s (MS), 12 spectra/s (MS/MS); isolation width (MS/MS): narrow (~1.3*m/z*); collision energy: -4.8 (offset) + 3.6 (slope); max. precursors/ cycle: 15; active exclusion: enabled, exclude after one spectrum, release after 0.25 min; charge state preference: 2, 3 and >3, sorted by abundance only; reference mass: *m/z* 299.294457 and 1221.990637 from continuous addition of trace amounts of methyl stearate and HP-1221 calibrant respectively into the ionization region. Reference mass correction was enabled.

Data analysis

Mass spectrometric data were processed and analyzed using the Peaks software, version 7.5 (Bioinformatics Solutions Inc.) for MS/MS-based identification and *de novo* sequencing. For protein identification, MS/MS spectra were searched against the Uniprot/Swissprot *Helicobacter pylori* (strain J99 / ATCC 700824) accepting common variable modifications with maximum three

variable PTM per peptide. The search parameters allowed for three trypsin missed cleavages per peptide, the precursor mass tolerance was set to 5 ppm, and fragment ion mass tolerance was set to 0.2 Da. The false discovery rate (FDR) was below <1% and this resulted in a -10log p-value of greater than 20.

Results

UniProtKB accession no.	Gene name	Protein name	Organism	Theoretical molecular weight (kDa)	-10logP	Coverage (%)	No. of peptides	No. of unique peptides	Frequency
Q9ZLL0	*ispG*	4-hydroxy-3-methylbut-2-en-1-yl diphosphate synthase (flavodoxin)	*Helicobacter pylori* (strain J99 / ATCC 700824)	39.26	62.42	36	11	11	1
Q9ZMD9	*rplU*	50S ribosomal protein L21	*Helicobacter pylori* (strain J99 / ATCC 700824)	11.84	31.02, 35.77	37, 27	3, 3	3, 3	2
Q9ZLS3	*accA*	Acetyl-coenzyme A carboxylase carboxyl transferase subunit alpha	*Helicobacter pylori* (strain J99 / ATCC 700824)	34.92	39.24	27	3	3	1
Q9ZL64	*acnB*	Aconitate hydratase 2	*Helicobacter pylori* (strain J99 / ATCC 700824)	92.74	103.39	25	19	19	1
Q9ZKH4	*speA*	Arginine decarboxylase	*Helicobacter pylori* (strain J99 / ATCC 700824)	70.32	82.36	23	9	9	1
Q9ZM66	*ftsH*	ATP-dependent zinc metalloprotease FtsH	*Helicobacter pylori* (strain J99 / ATCC 700824)	69.77	110.26	53	24	24	1

Q9ZK23	*rpoBC*	Bifunctional DNA-directed RNA polymerase subunit beta-beta'	*Helicobacter pylori* (strain J99 / ATCC 700824)	323.75	196.85, 189.73, 155.21	57, 39, 40	176,103, 96	176, 103, 96	3
Q9ZL53	*cadA*	Cadmium zinc and cobalt-transporting ATPase	*Helicobacter pylori* (strain J99 / ATCC 700824)	74.84	112.21	29	20	20	1
Q9ZLV3	*virD4*	Cag island protein DNA transfer protein	*Helicobacter pylori* (strain J99 / ATCC 700824)	85.79	101.47	31	17	16	1
Q9ZLU1	*cagM*	Cag island protein	*Helicobacter pylori* (strain J99 / ATCC 700824)	43.73	41.34, 82.71	21, 23	4, 5	4, 5	2
Q9ZLU0	*cagN*	Cag island protein	*Helicobacter pylori* (strain J99 / ATCC 700824)	34.74	46.94	22	4	4	1
Q9ZLU9	*orf15*	Cag island protein	*Helicobacter pylori* (strain J99 / ATCC 700824)	60.59	91.15	37	21	21	1
Q9ZM69	*copA*	Copper-transporting ATPase	*Helicobacter pylori* (strain J99 / ATCC 700824)	81.91	111.82	23	13	13	1
Q9ZKX3	*ruvC*	Crossover junction endodeoxyribonuclease RuvC	*Helicobacter pylori* (strain J99 / ATCC 700824)	17.43	37	21	2	2	1
Q9ZLT1	*cagA*	Cytotoxicity-associated immunodominant antigen	*Helicobacter pylori* (strain J99 / ATCC 700824)	129.73	96.24	20	17	17	1
Q9ZM99	*pyrG*	CTP synthase	*Helicobacter pylori* (strain J99 / ATCC 700824)	60.76	107.96	26	14	14	1
Q9ZMR8	*hemB*	Delta-aminolevulinic acid dehydratase	*Helicobacter pylori* (strain J99 / ATCC 700824)	36.25	79.96	42	11	11	1
Q9ZMD6	*dppB*	Dipeptide transport system permease protein	*Helicobacter pylori* (strain J99 / ATCC 700824)	36.89	78.22	44	14	14	1

Q9ZK04	*dld*	D-Lactate Dehydrogenase	*Helicobacter pylori* (strain J99 / ATCC 700824)	105.51	121.47	22	22	22	1
Q9ZLX3	*gyrB*	DNA gyrase subunit B	*Helicobacter pylori* (strain J99 / ATCC 700824)	87.44	85.52, 94.44, 101.38	26, 35, 25	23, 24, 17	23, 23, 17	3
Q9ZJE1	*rep*	DNA helicase	*Helicobacter pylori* (strain J99 / ATCC 700824)	77.59	112.69	44	30	30	1
Q9ZJF9	*dnaE*	DNA polymerase III subunit alpha	*Helicobacter pylori* (strain J99 / ATCC 700824)	138.04	126.58	28	35	34	1
Q9ZN49	*dnaG*	DNA primase	*Helicobacter pylori* (strain J99 / ATCC 700824)	63.82	96.46	43	21	21	1
Q9ZN44	*VirB4_1*	DNA transfer protein	*Helicobacter pylori* (strain J99 / ATCC 700824)	90.16	88.9	31	16	16	1
Q9ZMS6	*eno*	Enolase	*Helicobacter pylori* (strain J99 / ATCC 700824)	46.65	46.88	23	6	6	1
Q9ZMD0	*hemL*	Glutamate-1-semialdehyde 2 1-aminomutase	*Helicobacter pylori* (strain J99 / ATCC 700824)	46.73	34.08	30	4	3	1
Q9ZL09	*der*	GTPase Der	*Helicobacter pylori* (strain J99 / ATCC 700824)	51.75	41.64	23	5	5	1
Q9ZLZ3	*typA*	GTP-binding protein TypA/BipA homolog	*Helicobacter pylori* (strain J99 / ATCC 700824)	66.65	142.28	58	42	42	1
Q9ZJL3	*lon*	Lon protease	*Helicobacter pylori* (strain J99 / ATCC 700824)	93.95	117.3, 113.45	33, 21	33, 15	33, 15	2
Q9ZJA7	*selA*	L-seryl-Trna(Sec) selenium transferase	*Helicobacter pylori* (strain J99 / ATCC 700824)	44.00	42.56, 69.93	27, 35	4, 9	4, 9	2

Q9ZM79	*msbA*	Multi-drug resistance protein	*Helicobacter pylori* (strain J99 / ATCC 700824)	61.93	90.15	29	12	12	1
Q9ZKD8	*gdhA*	NADP-specific glutamate dehydrogenase	*Helicobacter pylori* (strain J99 / ATCC 700824)	49.49	29.36	22	3	3	1
Q9ZME2	*pabB*	P-amino benzoate synthetase	*Helicobacter pylori* (strain J99 / ATCC 700824)	65.48	76.58	23	11	11	1
Q9ZMZ0	*prfA*	Peptide chain release factor 1	*Helicobacter pylori* (strain J99 / ATCC 700824)	39.59	101.32	61	23	23	1
Q9ZMD7	*dppA*	Periplasmic dipeptide transport substrate-binding protein	*Helicobacter pylori* (strain J99 / ATCC 700824)	61.84	100.46	25	13	13	1
Q9ZJP1	*pgk*	Phosphoglycerate kinase	*Helicobacter pylori* (strain J99 / ATCC 700824)	44.71	103.67	37	16	16	1
Q9ZL11	*speE*	Polyamine aminopropyltransferase	*Helicobacter pylori* (strain J99 / ATCC 700824)	30.68	126	68	23	23	1
Q9ZJE3	*jhp_1369*	Probable aromatic acid decarboxylase	*Helicobacter pylori* (strain J99 / ATCC 700824)	20.60	25.71	22	2	2	1
Q9ZN12	*putA*	Proline/pyrroline-5-carboxylate dehydrogenase	*Helicobacter pylori* (strain J99 / ATCC 700824)	135.23	89.26	21	16	15	1
Q9ZK86	*tolB*	Protein TolB	*Helicobacter pylori* (strain J99 / ATCC 700824)	47.74	80.1, 79.6	23, 41	7, 13	7, 13	2
Q9ZMV5	*jhp_0110*	Putative	*Helicobacter pylori* (strain J99 / ATCC 700824)	47.12	85.51, 92.87	46, 50	18, 20	17, 19	2
Q9ZMA6	*jhp_0316*	Putative	*Helicobacter pylori* (strain J99 / ATCC 700824)	29.89	44.44, 83.67	31, 55	5, 10	5, 10	2

Q9ZKK9	*jhp_0926*	Putative	*Helicobacter pylori* (strain J99 / ATCC 700824)	39.49	78.63, 112.09	42, 62	17, 35	17, 35	2
Q9ZKK7	*jhp_0928*	Putative	*Helicobacter pylori* (strain J99 / ATCC 700824)	255.77	160.03, 171.65	39, 34	79, 78	78, 78	2
Q9ZJN3	*JHP1272*	Putative	*Helicobacter pylori* (strain J99 / ATCC 700824)	134.38	107.26, 117.52	34, 39	29, 38	2, 38	2
Q9ZN33	*jhp_0026*	Putative	*Helicobacter pylori* (strain J99 / ATCC 700824)	64.87	89.22	32	18	18	1
Q9ZMZ9	*jhp_0061*	Putative	*Helicobacter pylori* (strain J99 / ATCC 700824)	92.77	116.92	33	28	28	1
Q9ZMJ3	*jhp_0226*	Putative	*Helicobacter pylori* (strain J99 / ATCC 700824)	15.43	49.06	37	4	4	1
Q9ZMF1	*jhp_0269*	Putative	*Helicobacter pylori* (strain J99 / ATCC 700824)	60.02	103.15	39	19	19	1
Q9ZLY5	*jhp_0440*	Putative	*Helicobacter pylori* (strain J99 / ATCC 700824)	103.46	176.05	69	100	100	1
Q9ZLX2	*jhp_0454*	Putative	*Helicobacter pylori* (strain J99 / ATCC 700824)	39.89	123.58	28	10	10	1
Q9ZLN6	*jhp_0542*	Putative	*Helicobacter pylori* (strain J99 / ATCC 700824)	55.24	88.31	48	23	23	1
Q9ZLL3	*jhp_0566*	Putative	*Helicobacter pylori* (strain J99 / ATCC 700824)	13.30	20.34	31	1	1	1
Q9ZLB2	*jhp_0668*	Putative	*Helicobacter pylori* (strain J99 / ATCC 700824)	66.03	99.36	37	18	18	1

Q9ZL70	*jhp_0710*	Putative	*Helicobacter pylori* (strain J99 / ATCC 700824)	39.52	68.41	42	11	11	1
Q9ZKU3	*jhp_0842*	Putative	*Helicobacter pylori* (strain J99 / ATCC 700824)	57.93	61.1	21	6	6	1
Q9ZKH0	*jhp_0966*	Putative	*Helicobacter pylori* (strain J99 / ATCC 700824)	38.55	147.17	61	29	29	1
Q9ZJU4	*jhp_1204*	Putative	*Helicobacter pylori* (strain J99 / ATCC 700824)	39.82	43.07	21	3	3	1
Q9ZJN5	*jhp_1270*	Putative	*Helicobacter pylori* (strain J99 / ATCC 700824)	34.63	89.61	46	12	12	1
Q9ZJE0	*jhp_1372*	Putative	*Helicobacter pylori* (strain J99 / ATCC 700824)	96.43	104.92	27	24	22	1
Q9ZKR0	*jhp_0875*	Putative amino acid ABC transporter binding protein	*Helicobacter pylori* (strain J99 / ATCC 700824)	28.78	34.6	26	3	3	1
P64102	*jhp_1339*	Putative biopolymer transport protein ExbD-like 2	*Helicobacter pylori* (strain J99 / ATCC 700824)	15.04	32.57	29	2	2	1
Q9ZJB2	*jhp_1401*	Putative ferredoxin-like protein	*Helicobacter pylori* (strain J99 / ATCC 700824)	52.77	102.74	41	23	23	1
Q9ZM95	*fliH*	Putative flagellar export apparatus	*Helicobacter pylori* (strain J99 / ATCC 700824)	29.17	69.53	51	13	13	1
Q9ZL17	*jhp_0765*	Putative lipopolysaccharide biosynthesis protein	*Helicobacter pylori* (strain J99 / ATCC 700824)	31.62	23.64	22	2	2	1
Q9ZMJ4	*ispB*	Putative octaprenyl-diphosphate synthase	*Helicobacter pylori* (strain J99 / ATCC 700824)	35.03	75.02	44	13	13	1

Q9ZJI1	*sppA*	Putative protease IV	*Helicobacter pylori* (strain J99 / ATCC 700824)	32.51	59.07	34	6	5	1
Q9ZM14	*jhp_0409*	Putative short chain dehydrogenase	*Helicobacter pylori* (strain J99 / ATCC 700824)	28.53	67.32	31	9	8	1
Q9ZML8	*jhp_0200*	Putative transporter	*Helicobacter pylori* (strain J99 / ATCC 700824)	59.44	80.29	24	10	10	1
Q9ZKE0	*jhp_0999*	Putative zinc-metallo protease	*Helicobacter pylori* (strain J99 / ATCC 700824)	46.37	73.48	40	13	13	1
Q9ZJX9	*rnr*	Ribonuclease	*Helicobacter pylori* (strain J99 / ATCC 700824)	74.16	89.35	38	22	22	1
Q9ZK65	*rimM*	Ribosome maturation factor RimM	*Helicobacter pylori* (strain J99 / ATCC 700824)	20.40	82.89	42	7	7	1
Q9ZJH2	*smpB*	SsrA-binding protein	*Helicobacter pylori* (strain J99 / ATCC 700824)	17.81	46.99	33	3	3	1
Q9ZMV3	*thrS*	Threonine—Trna ligase	*Helicobacter pylori* (strain J99 / ATCC 700824)	70.16	81.46	31	17	17	1
Q9ZJ57	*mfd*	Transcription-repair-coupling factor	*Helicobacter pylori* (strain J99 / ATCC 700824)	113.44	92.62	24	18	18	1
Q9ZJA6	*nusA*	Transcription termination/antitermination protein NusA	*Helicobacter pylori* (strain J99 / ATCC 700824)	44.74	87.71, 110.35	42, 46	14, 17	14, 17	2
Q9ZM46	*infB*	Translation initiation factor IF-2	*Helicobacter pylori* (strain J99 / ATCC 700824)	105.92	81.3	21	15	15	1
Q9ZML9	*mnmG*	Trna uridine 5-	*Helicobacter pylori* (strain	69.69	79.12	31	15	15	1

		carboxymethylaminomethyl modification enzyme MnmG	J99 / ATCC 700824)						
Q9ZJ90	*hsdR_3*	Type I restriction enzyme (restriction subunit)	*Helicobacter pylori* (strain J99 / ATCC 700824)	115.81	100.94	37	30	30	1
Q9ZJN4	*jhp_1271*	Type II DNA modification enzyme (methyltransferase)	*Helicobacter pylori* (strain J99 / ATCC 700824)	41.91	75.18	39	13	13	1
V6CKT1	*JHP1272*	Type IIG R-M system	*Helicobacter pylori* (strain J99 / ATCC 700824)	124.27	106.47	35	29	2	1
Q9ZKY6	*coaX*	Type III pantothenate kinase	*Helicobacter pylori* (strain J99 / ATCC 700824)	24.75	46.21	31	3	3	1
Q9ZJA3	*res_2*	Type III restriction enzyme	*Helicobacter pylori* (strain J99 / ATCC 700824)	111.67	140.57	50	49	48	1
Q9ZMN6	*lpxD*	UDP-3-O-acylglucosamine N-acyltransferase	*Helicobacter pylori* (strain J99 / ATCC 700824)	36.56	28.87	23	2	2	1
P64654	*jhp_0112*	Uncharacterized protein	*Helicobacter pylori* (strain J99 / ATCC 700824)	4.92	36.68	35	1	1	1
Q9ZL98	*jhp_0380*	Uncharacterized metallophosphoesterase	*Helicobacter pylori* (strain J99 / ATCC 700824)	42.17	91.16	41	8	8	1
Q9ZM43	*jhp_0682*	Uncharacterized RNA pseudouridine synthase	*Helicobacter pylori* (strain J99 / ATCC 700824)	37.72	140.77	64	39	39	1
Q9ZLR4	*jhp_0514*	UPF0118 membrane protein	*Helicobacter pylori* (strain J99 / ATCC 700824)	39.81	92.21	36	12	12	1
